# ATBS1-INTERACTING FACTOR 2 negatively regulates dark- and brassinosteroid-induced leaf senescence through interactions with INDUCER OF CBF EXPRESSION 1

**DOI:** 10.1093/jxb/erz533

**Published:** 2019-11-30

**Authors:** Yoon Kim, Seon-U Park, Dong-Min Shin, Giang Pham, You Seung Jeong, Soo-Hwan Kim

**Affiliations:** 1 Division of Biological Science and Technology, Yonsei University, Wonju-Si, Republic of Korea; 2 University of Birmingham, UK

**Keywords:** Arabidopsis, ATBS1-INTERACTING FACTOR 2 (AIF2), basic helix–loop–helix (bHLH), brassinosteroid (BR), C-REPEAT BINDING FACTOR (CBF), INDUCER OF CBF EXPRESSION 1 (ICE1), leaf senescence, PHYTOCHROME-INTERACTING FACTORS (PIFs)

## Abstract

ATBS1-INTERACTING FACTOR 2 (AIF2) is a non-DNA-binding basic helix–loop–helix (bHLH) transcription factor. We demonstrated that AIF2 retards dark-triggered and brassinosteroid (BR)-induced leaf senescence in *Arabidopsis thaliana*. Dark-triggered BR synthesis and the subsequent activation of BRASSINAZOLE RESISTANT 1 (BZR1), a BR signaling positive regulator, result in BZR1 binding to the AIF2 promoter in a dark-dependent manner, reducing *AIF2* transcript levels and accelerating senescence. BR-induced down-regulation of AIF2 protein stability partly contributes to the progression of dark-induced leaf senescence. Furthermore, AIF2 interacts with INDUCER OF CBF EXPRESSION 1 (ICE1) via their C-termini. Formation of the AIF2–ICE1 complex and subsequent up-regulation of *C-REPEAT BINDING FACTOR*s (*CBF*s) negatively regulates dark-triggered, BR-induced leaf senescence. This involves antagonistic down-regulation of *PHYTOCHROME INTERACTING FACTOR 4* (*PIF4*), modulated through AIF2-dependent inhibition of ICE1’s binding to the promoter. PIF4-dependent activities respond to dark-induced early senescence and may promote BR synthesis and BZR1 activation to suppress AIF2 and accelerate dark-induced senescence. Taken together, these findings suggest a coordination of AIF2 and ICE1 functions in maintaining stay-green traits.

## Teaser

AIF2–ICE1 transcriptional complex is negatively involved in dark-triggered and brassinosteroid-induced leaf senescence, maintaining plant growth and enabling them to stay green longer.

## Introduction

Aging or leaf senescence is the final stage of leaf development. Nutrients and energy from leaves are redirected to new and developing tissues or storage organs. Senescence involves substantial metabolic reprogramming including chlorophyll degradation, protein degradation, reallocation of nutrients, increase in reactive oxygen species (ROS), enhanced programmed cell death/necrosis, membrane ion leakage, and differential expression of numerous genes, including senescence-associated genes (SAGs) (Fischer, 2012; Sarwat *et al.*, 2013; Havé *et al.*, 2017). Many developmental and environmental signals such as age, drought, lack of nutrients, oxidative and temperature stresses, and pathogen attack influence the onset of leaf senescence in plants (Woo *et al.*, 2013). Hormones are endogenous factors regulating the onset and progression of leaf senescence, mostly by relaying environmental changes to the cell machinery. Cytokinin (CK; Zwack *et al.*, 2013) and auxins (Cha *et al.*, 2016) delay senescence, whereas ethylene (Koyama, 2014), jasmonic acid (JA; Hu *et al.*, 2017), abscisic acid (ABA; Gao *et al.*, 2016), gibberellin (Chen *et al.*, 2017), and salicylic acid (SA; Zhang *et al.*, 2013) accelerate leaf senescence. Light deprivation in the form of severe shading and darkening of leaves also accelerates senescence. PHYTOCHROME-INTERACTING FACTORS (PIFs) orchestrate dark-induced senescence (DIS) by targeting chloroplast maintenance, chlorophyll metabolism, hormone signaling, and expression of senescence master regulators, suggesting potential molecular links to the energy deprivation signaling pathway (Liebsch and Keech, 2016). PIF3, PIF4, and PIF5 promote age-triggered and dark-induced leaf senescence (Song *et al.*, 2014). Specifically, PIF4 negatively regulates chloroplast activity and decreases dark-induced ethylene biosynthesis and ethylene-induced leaf senescence.

Brassinosteroids (BRs) are plant steroid hormones that play crucial roles in plant growth and development via extensive signal integration through direct interactions with numerous signaling pathways (Belkhadir and Jaillais, 2015). Brassinolide (BL) is the most active BR, and its production is tightly regulated by a coordinative feedback regulation of key biosynthetic enzymes and their catalytic reactions (Zhao and Li, 2012). These reactions include DE-ETIOLATED 2 (DET2) converting the sterol progenitor campesterol to campestanol, DWARF4 (DWF4) and CONSTITUTIVE MORPHOGENESIS (CPD) hydroxylating steroid intermediates at a C-22 or C-23 position, and BR6 oxidase (BR6ox) producing BL from deoxoteasterone and castasterone. Recently, BR perception and signal transduction have been characterized in detail. Upon binding of BRs to BRASSINOSTEROID INSENSITIVE 1 (BRI1), a complex of BRI1 and its binding partner BRI1-ASSOCIATED RECEPTOR KINASE 1 (BAK1) initiates a signaling cascade, relaying the membrane surface signal to the nucleus to activate the positively acting transcription factors BRASSINAZOLE RESISTANT 1/BRI1 EMS SUPPRESSOR 2 (BZR1/BES2) and BZR2/BES1 to regulate expression of numerous genes involved in plant growth and development (He *et al.*, 2005; Sun *et al.*, 2010). Their growth-promoting signaling pathways are positively regulated via BZR1/BES1-mediated signaling events, and they are balanced with negative signaling modules transmitted through GSK3/SHAGGY-LIKE BRASSINOSTEROID-INSENSITIVE 2 (ATSK21/BIN2) and other transcription factors, such as ATBS1-INTERACTING FACTOR 2 (AIF2) (Belkhadir and Jaillais, 2015; Kim *et al.*, 2017). AIF2 is a BIN2-interacting and non-DNA-binding bHLH protein, and BIN2-driven AIF2 phosphorylation augments the BIN2/AIF2-mediated negative circuit of BR signaling pathways in growth regulation. In contrast, BR-induced transcriptional repression and protein degradation negatively regulate transcription factor AIF2, reinforcing the BZR1/BES1-mediated positive BR signaling pathway (Kim *et al.*, 2017).

BRs are generally known as positive regulators of senescence. For example, a BR biosynthesis mutant, *de-etiolated 2* (*det2*), displays delayed leaf yellowing (Chory *et al.*, 1991). Ectopic expression of BAK7 (a BAK1 homologue) partially rescued the *bri1* (a BR receptor mutant) phenotype, and introduction of an RNAi construct of *BAK7* in Arabidopsis resulted in severe growth retardation and early senescence (Jeong *et al.*, 2010). High-throughput RNA sequencing analysis revealed that three BR-related genes encoding BAK1, BR6ox, and a BAK1 precursor were differentially expressed in early and late-senescence cotton lines (Kong *et al.*, 2013). It was also demonstrated in Arabidopsis that four senescence-associated genes identified from senescence-promoting enhancer trap-lines were significantly up-regulated by exogenous application of BR (He *et al.*, 2001). Although phenotypic effects of BR treatment and BR-related genetic mutation on plant senescence have been reported, there is a knowledge gap regarding BR action in plant senescence. Molecular signaling mechanisms and proteins associated with their regulation are particularly unclear.

INDUCER OF CBF EXPRESSION 1 (ICE1) encodes a nuclear-localized MYC-like bHLH transcription factor; its dominant negative mutant, *ice1*, impairs chilling and freezing tolerance (Chinnusamy *et al.*, 2003). *C-REPEAT BINDING FACTORs CBF1, CBF2, and CBF3* are well-known ICE1 transcriptional targets that regulate cold tolerance by inducing expression of diverse cold responsive (*COR*) genes (Shi *et al.*, 2017). In this study, we identified transcription factor AIF2 as a positive retardation regulator of age- and dark-induced leaf senescence. We also provided molecular evidence demonstrating that BZR1-mediated BR signals promote leaf senescence by suppressing AIF2 both transcriptionally and post-translationally. The successful formation of an AIF2–ICE1 complex, up-regulation of *CBF*s, and the antagonistic down-regulation of *PIF4* are negatively involved in dark-triggered and BR-induced leaf senescence, maintaining plant growth and enabling them to stay green longer.

## Materials and methods

### Plant material and growth conditions

Wild-type *Arabidopsis thaliana* (Columbia-0, Col-0) and its BR-related mutants (*bzr1-1D*, *det2*), AIF2 T-DNA knockout plant (*aif2-1*, CS811403), AIF2-overexpressing *p35S::AIF2-EGFP* transgenic plants (AIF2ox), β-glucuronidase (GUS)-expressing *pAIF2::AIF2-GUS* (Kim *et al.*, 2017), and *Arabidopsis thaliana* (Wassilewskija-2, Ws-2) and its loss-of-function mutant of BR receptor BRI1 (*bri1-5*) (Noguchi *et al.*, 1999) were used for gene/reporter expression and phenotype analyses. Seeds of lines carrying *pBZR1::BZR1-EGFP* and *p35S::bzr1-1D-GFP* were kindly provided by Dr Z. Y. Wang (Carnegie Institution for Science, USA). Seeds of a line carrying *35S::BIN2-myc* were provided by Dr T.-W. Kim (Hanyang University, South Korea). Following 2 d of cold stratification, seeds were grown in pots containing Sunshine No. 5 soil (Polysciences) in an environmentally controlled growth room under a cycle of 16 h light (100–150 µmol m^−2^ s^−1^) and 8 h dark at 23–25 °C with 80–85% humidity.

### *In vivo* interaction tests in yeast

All procedures for yeast transformation and protein interaction were performed according to Clontech’s Yeast Protocols Handbook (PT3024-1) using *pGBKT7* as the GAL4 DNA-binding domain vector and *pGADT7* as the GAL4 DNA activation vector (Clontech, USA). Briefly, cDNAs encoding a full-length or truncated forms of *AIF2* (At3g06590), *ICE1* (At3g26744), and *BIN2* (At4g18710) (depicted in Fig. 4A) were PCR-amplified (see Supplementary Table S1 at *JXB* online) and subsequently inserted into either *pGBKT7* or *pGADT7* vector to generate constructs testing interactions between AIF2 and ICE1 in yeast cells (*Saccharomyces cerevisiae* AH109). These plasmids were co-transformed into yeast cells of equal densities. The transformed yeast cells were then quantitatively analysed for interactions between test proteins by measuring β-galactosidase activity, determined using chlorophenol red-β-D-galactopyranoside as a substrate.

### Generation of binary constructs, co-immunoprecipitation, and bimolecular fluorescence complementation assays in tobacco

To test protein interactions *in planta* using co-immunoprecipitation assays, cDNAs encoding either full-length or truncated forms of *AIF2*, *ICE1*, and *BIN2* (Fig. 4A) were PCR-amplified (see Supplementary Table S1) and cloned into *pGWB20* in fusion with myc (Nakagawa *et al.*, 2007). Next, a combination of *Agrobacterium tumefaciens* GV301 containing *p35S::AIF2FL-nYFL*, *p35S::BIN2-myc*, or different binary deletion constructs of ICE1 (*p35S::ICE1FL-myc*, *p35S::ICE1dN-myc*, *p35S::ICE1dC-myc*) was co-infiltrated through the underside of 2- to 4-week-old tobacco (*Nicotiana benthamiana*) leaves. Total proteins were then extracted from the infiltrated leaves, and immunoprecipitation was performed using anti-myc antibody (Abcam, UK)-conjugated Protein G magnetic beads (Bio-Rad, USA). Co-immunoprecipitated proteins were then separated using 12% SDS-PAGE, and the presence of AIF2FL–nYFP and BIN2–myc or ICE1–myc proteins was analysed by immunoblotting with anti-green fluorescent protein (GFP) antibodies or anti-myc antibodies (Santa Cruz Biotechnology, USA), respectively. For an *in planta* interaction assay by bimolecular fluorescence complementation (BiFC), a combination of *Agrobacterium* containing full-length or part-length coding regions of *AIF2*, *ICE1*, and *BIN2* in *pPZP312-nYFP* or *pPZP312-cCFP*, in fusion with the N-terminal half of YFP (nYFP) or the C-terminal half of CFP (cCFP), respectively (Gampala *et al.*, 2007) (*p35S::ICE1FL-nYFP*, *p35S::AIF2FL-cCFP*, *AIF2dN-cCFP*, *p35S::AIF2dC-cCFP*, *pVec-cCFP*, *p35S::AIF2FL-nYFP*, *p35S::BIN2-cCFP*, *p35S::ICE1FL-cCFP*, *p35S::ICE1dC-cCFP*, and *pVec-nYFP*) was co-infiltrated into the underside of 4-week-old tobacco leaves. After 36–48 h of incubation, epidermal cell layers were observed for fluorescence using the Zeiss LSM 710 confocal laser scanning microscope. 4′,6-Diamidino-2-phenylindole was used as the marker for indicating nuclear position.

### Generation of transgenic plants

For generation of transgenic plants ectopically expressing variants of AIF2–EGFP and ICE1–myc, cDNAs encoding either the full-length or truncated form of ICE1 or AIF2 were inserted upstream of the myc-tag-expressing *pGWB20* (Nakagawa *et al.*, 2007) or EGFP-expressing *pB7FWG2* (Karimi *et al.*, 2005) binary vectors. Subsequently, *Agrobacterium* cultures carrying each construct was introduced to transform *bzr1-1D*, Col-0, or *aif2-1* genetic lines using the floral dipping method (Clough and Bent, 1998), generating plants designated as *p35S::AIF2FL-EGFP*/*bzr1-1D*, *p35S::AIF2dC-EGFP*/*bzr1-1D*, *p35S::ICE1FL-myc*/Col-0, *p35S::ICE1dC-myc*/Col-0, *p35S::ICE1FL-myc*/*aif2-1*, and *p35S::ICE1dC-myc*/*aif2-1*. Expression of EGFP- or myc-tagged transgenic proteins was confirmed by western blot analysis using anti-GFP or -myc antibodies as probes.

### Total RNA isolation, quantitative RT-PCR, and chromatin immunoprecipitation-qPCR assay

All procedures are previously described (Kim *et al.*, 2017). Quantitative RT-PCR (qRT-PCR) was performed with primers (see Supplementary Table S2) amplifying the indicated genes. The chromatin immunoprecipitation (ChIP)-qPCR experiments were performed using non-transgenic plants and plants expressing a native promoter-driven BZR1-GFP (*pBZR1::BZR1-EGFP*; Fig. 2D) or expressing CaMV35S-driven ICE1FL or ICE1dC (*p35S::ICE1FL-myc* or *p35S::ICE1dC-myc*, respectively; Fig. 6E, I). The amounts of BZR1- or ICE1FL-/ICE1dC-bound DNAs from the tested genes were measured by real-time PCRs with primer sets (Supplementary Table S3) using equal amounts of DNA from the input and co-immunoprecipitation fractions.

### Staining and measurements of autofluorescence, chlorophyll content, and membrane ion leakage

Trypan blue (TB) and diaminobenzidine (DAB) staining were performed as described previously (Hirashima *et al.*, 2009). After TB or DAB staining and clearing of chlorophyll, samples were observed under a differential interference contrast microscope. The chlorophyll content was measured as described previously with minor modifications (Arnon, 1949). To measure membrane ion leakage (Li *et al.*, 2013), leaves were incubated in deionized water for 2 h, and the conductivities (C1) of the solutions were determined. The leaves were subsequently boiled in the same deionized water for 15 min, and the conductivities (C2) of the resulting solutions were re-measured. The ratio of C1:C2 was calculated and used to evaluate the degree of electrolyte leakage. Autofluorescence images of plant leaves were captured using a fluorescence imaging system (Neoscience, South Korea). Images were quantitatively analysed using NEOimage software.

### Luciferase and β-glucuronidase reporter assays

To examine effector-induced transient transcription of the *CBF2* promoter- or *PIF4* promoter-driven luciferase reporter (*pCBF2::Luc and pPIF4::Luc*, respectively) *in planta*, a genomic DNA fragment covering 1292 or 1236 bp upstream of the *CBF2* (AT4G25470) or *PIF4* (AT2G43010) transcription start site, respectively, was PCR-amplified (see Supplementary Table S1) and cloned in Gateway-compatible binary vector *pBGWL7* in fusion with the full-length coding region of the luciferase gene (Karimi *et al.*, 2005; https://gateway.psb.ugent.be). Next, *Agrobacterium tumefaciens* GV301 carrying *pCBF2::LuC* or *pPIF4::Luc* together with *Agrobacterium* containing different combinations of AIF2 and ICE1 effectors was co-infiltrated, along with the transfection control (*p35S::GUS*), through the underside of 2- to 4-week-old tobacco leaves. After 36–48 h of incubation in the dark, luciferase activity in the transfected leaves was measured using a GloMax 20/20 luminometer (Promega, USA). Data regarding luciferase activity were normalized to GUS activity of the same transfected samples. The GUS activity in leaves of transiently transformed tobacco and *pAIF2::AIF2-GUS*-expressing plants was measured according to a previously described method (Blázquez, 2007).

### Senescence-related transcriptome profile analysis

To compare senescence-related global gene expression in *ice1* and Col-0 plants undergoing dark-induced senescence, we first retrieved a transcriptome profile representing gene expression in *ice1* (Lee *et al.*, 2005; a GEO dataset GSE3326, https://www.ncbi.nlm.nih.gov/gds) and compared it with the genes differentially expressed in dark-induced Col-0 plants (van der Graaff *et al.*, 2006). The differentially expressed genes (DEGs) in the *ice1* mutant were identified using the R program (version 3.5.2), and the edgeR package was used for normalization and correction within arrays (Robinson *et al.*, 2010; McCarthy *et al.*, 2012). The false discovery rate (FDR)-adjusted *P* value was below 0.05, and expression level 1<log_2_(fold change)<−1 was applied to identify significantly regulated DEGs. A list of PIF4- or BZR1-targeted genes was obtained from chromatin-immunoprecipitation sequencing (ChIP-Seq) analysis (Sun *et al.*, 2010; Oh *et al.*, 2012), which was used to identify overlapping genes related to senescence- and *ice1*-regulated genes. Venn diagrams were constructed using an embed function of R, and heatmaps were generated using gplots (https://cran.r-project.org/web/packages/gplots/gplots.pdf). Gene ontology (GO) annotation and enrichment of GO terms were performed using resources from The Gene Ontology Consortium (http://www.geneontology.org/; Ashburner *et al.*, 2000; The Gene Ontology Consortium et al., 2016), and the GO categories significantly enriched with FDR *P*-value <0.05 were identified.

### Measurement and statistical analysis

More than 30 fourth leaves were collected from 5-week-old plants and used for each experiment described in ‘Results’. All experiments were conducted at minimum in triplicate, and the data were statistically analysed by Student’s *t*-test.

## Results

### AIF2 negatively influences both dark-induced and age-dependent leaf senescence

**Fig. 1.**
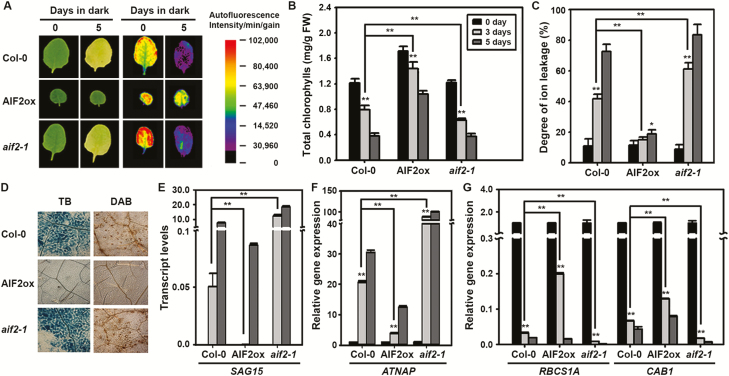
Dark-induced progression of leaf senescence in Col-0, AIF2ox, and *aif2-1* plants. The fourth leaves of 5-week-old Col-0, AIF2ox, and *aif2-1* plants were subjected to dark for 3 or 5 d. Seen above are visible leaf phenotypes and their autofluorescence (A), degree of senescence measured by total chlorophyll amount (B), and degree of ion leakage (C) before and after dark incubation. Leaves incubated in dark for 5 d were stained with TB and DAB (D). Transcript accumulation of senescence-related genes was examined using qRT-PCR (E–G). Values in (F) and (G) were normalized to those of 0-day plants, which were set to 1. Bar graphs represent means ±SD. Asterisks on bars indicate statistical difference from the 0-day sample of each Col-0, AIF2ox, and *aif2-1* plant; asterisks on bracketed samples represent a statistical difference between the two compared samples; *P<0.05, **P<0.01. (This figure is available in color at *JXB* online.)

Previously, we reported that ectopic expression of *AIF2* in Arabidopsis (*p35S::AIF2-EGFP*, AIF2ox) produced dwarfed and greener plants, a phenotype typical of BR biosynthetic- or signaling-defective plants (Kim *et al.*, 2017). We therefore decided to systematically compare the dark-induced senescence phenotypes observed in leaves of wild type Col-0, AIF2ox, and the loss-of-function mutant *aif2-1*. Darkness induces rapid and synchronous senescence in detached leaves, and such a leaf system has been used as a senescence model to study age- and development-triggered senescence (Weaver and Amasino, 2001). Dark-incubated leaves of Col-0 plants experienced dramatic senescence as seen by fewer intact chloroplasts, represented by blue and purple autofluorescence after 5 d of dark incubation (Fig. 1A). Significant reduction in chlorophyll content (>30% reduction by 3 d of incubation and 55% reduction by 5 d of incubation) supports this finding (Fig. 1B). Moreover, these dark-treated plants lost ions rapidly (Fig. 1C), indicating cell death. Interestingly, these senescence phenotypes were further exacerbated in *aif2-1* loss-of-function plants. AIF2ox plants, however, lost fewer chloroplasts (green to orange autofluorescence), less chlorophyll, and fewer ions from their leaves. TB staining visualizes cell death, while DAB staining reveals the presence of ROS such as hydrogen peroxide and superoxide. Differential outcomes of senescence phenotypes were reconfirmed by extensive leaf staining by TB and DAB in Col-0 and *aif2-1* plants compared with that in AIF2ox plants (Fig. 1D). *SAG12* and *NAC-LIKE*, *ACTIVATED BY AP3/PI* (*AtNAP*) were highly up-regulated or acted as a positive regulator during senescence (Pontier *et al.* 1999; Guo and Gan, 2006). Comparing 3-day dark-incubated plants, we observed relatively higher up-regulation of *SAG12* and *AtNAP* transcripts in Col-0 and *aif2-1* plants than in AIF2ox plants (Fig. 1E, F, respectively). In comparison, *CHLOROPHYLL A/B BINDING PROTEIN 1* (*CAB1*) and *RIBULOSE BISPHOSPHATE CARBOXYLASE SMALL CHAIN 1A* (*RBCS1A*), two genes involved in active photosynthesis, were relatively highly expressed (less repressed compared with the 0-day incubated plants) in AIF2ox compared with those in Col-0 and *aif2-1* plants (Fig. 1G). The similar retardation of age-dependent senescence was observed in AIF2-overexpressing leaves (see Supplementary Fig. S1). AIF2ox plants showed fewer yellow-colored fourth leaves even at 60 d after germination (DAG) and lost fewer chlorophyll and ions from their leaves. Collectively, we concluded that AIF2 negatively influences both dark-induced and age-dependent leaf senescence.

### BZR1-mediated active BR signals occurring during onset and progression of dark periods result in decrease of AIF2 transcript and protein levels and the subsequent promotion of dark-induced leaf senescence

Hormones are major endogenous factors regulating onset and progression of leaf senescence (Sarwat *et al.*, 2013). Our results showed that BL (a BR) promoted leaf senescence in a concentration-dependent manner as determined by a decrease in total chlorophyll content (see Supplementary Fig. S2A) and an increase in membrane ion leakage (Supplementary Fig. S2B). As expected, *bzr1-1D* plants, with constitutive activation of the BR signaling pathway, showed further enhanced senescence phenotypes (Supplementary Fig. S2C, D). In contrast, the leaves of *det2* and *bri1-5* (a BR biosynthetic or signaling mutant, respectively) stayed relatively green and had increased longevity compared with their respective wild-type counterparts, Col-0 and Ws-2. Overall, these results demonstrate that BR and its BZR1-mediated signaling pathways exhibit positive roles in dark-triggered leaf senescence.

**Fig. 2.**
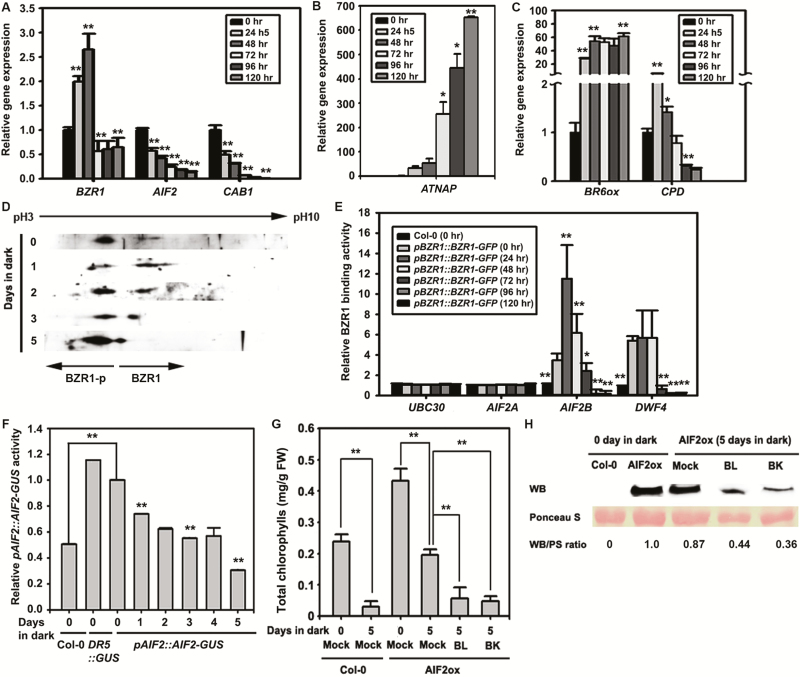
Dark-induced expression of BR- and senescence-related genes and BR-regulated *AIF2* transcription and protein stability. (A–C) Dark-induced and time-dependent expression of *AIF2* and BR biosynthesis genes in the fourth leaves of 5-week-old Col-0 plants. (D) Dark-induced BZR1 dephosphorylation/phosphorylation status in *pBZR1::BZR1-EGFP* plants, determined by two-dimensional SDS-PAGE followed by western blot analysis (Kim *et al.*, 2017). (E) ChIP-qPCR analysis of dark-dependent BZR1-binding activity to the *AIF2* gene promoter. The promoter of *UBC30* was used as a negative control for BZR1-binding activity. Transcript accumulation of genes presented in (A–C) or BZR1-binding to promoters in (E) was normalized to that of 0 h dark control of Col-0 plants, which was set to 1. (F) Time-dependent AIF2–GUS expression activity in dark-triggered *pAIF2::AIF2-GUS* plants. GUS activity was normalized to that of 0-day dark control of *pAIF2::AIF2-GUS* plants, which was set to 1. (G) Effects of brassinosteroid (10^−8^ M) and BK (10^−7^ M) on dark-triggered leaf senescence, determined by total chlorophyll accumulation in leaves. (H) Effects of BL and BK on AIF2 stability. Total proteins were extracted from leaves as described in (G). Western blot analysis was performed as previously described (Kim *et al.*, 2017). Ponceau S staining was used as a protein loading control. The normalized protein level (WB/PS) was set to 1 for *p35::AIF2-EGFP* plants incubated for 0 d in dark. Bar graphs represent means ±SD. An asterisk on bars indicates a statistical difference from the 0 hr sample of each gene (A–C) or *pBZR1::BZR1-GFP* plant (E) or from the 0-day sample of *pAIF2::AIF2-GUS plant* (F); asterisks on bracketed samples represent a statistical difference between the two compared samples; *P<0.05, **P<0.01. (This figure is available in color at *JXB* online.)

Our results so far demonstrate that exogenous BR treatment and ectopic expression of *AIF2* in leaves have opposite effects on age- and dark-induced senescence. Interestingly, the *AIF2* transcript level in the dark-induced leaves of Col-0 plants gradually declined in a time-dependent manner (Fig. 2A). Expression of a senescence-delaying marker, *CAB1*, decreased during senescence progression, while a senescence-promoting marker, *AtNAP*, increased dramatically (Fig. 2A, B). Expression of BR biosynthetic genes, *BR6ox* and *CPD*, was highly up-regulated (more than 30 times for *BR6ox* and 5.1 times for *CPD*) during the initial period of dark induction (24 h), and the expression level of *BR6ox* continued to increase thereafter (Fig. 2C). Similarly, expression of BZR1 gradually increased until 48 h after incubation in the dark but decreased thereafter (Fig. 2A). These results imply that the dark-promoted expression of *BR6ox* and *CPD* may result in high levels of endogenous BRs in leaves early in the process of initiating and undergoing senescence.

BZR1 and BZR2/BES1, which bind to BR response elements (BRREs, CGTGT/CG) and/or the E-box (CANNTG) of target genes, function as both a transcriptional activator and a repressor, promoting transcription of growth-promoting genes, while repressing genes involved in BR biosynthesis such as *CPD* and *DWF4* (He *et al.*, 2005; Sun *et al.*, 2010). Since the transcript levels of BR biosynthetic genes and *AIF2* are differently regulated during senescence, we hypothesized that the decreased *AIF2* transcript level during dark incubation might be attributed to higher endogenous BRs and the resulting enhanced positive BR signaling or stronger BZR1 activity. ATSK21/BIN2, a GSK3-like kinase, phosphorylates BZR1 to result in its degradation, and active BR signaling pathways, through KIB-mediated BIN2 degradation, lead to dephosphorylation and nuclear accumulation of BZR1 (He *et al.*, 2002; Ryu *et al.*, 2007; Zhu *et al.*, 2017). To assess the phosphorylation status of BZR1, we separated total proteins of dark-incubated *pBZR1::BZR1-GFP* plants by SDS-PAGE and observed changes in BZR1 localization along the isoelectric focusing (IEF) point. Phosphorylation of proteins makes them more basic, causing the phosphorylated forms of proteins to migrate toward the more acidic area (toward pH 3) on an IEF strip. Interestingly, 1 or 2 d of dark incubation led BZR1 protein to migrate toward the basic area (toward pH 10) of the IEF strip (Fig. 2D). This implies that the onset and early progression of dark incubation cause BZR1 protein to become more dephosphorylated than the 0 d-incubated control. In contrast, 5 d of dark incubation shifted the location of BZR1 toward the acidic area of the strip, signifying that longer dark incubation of plants renders BZR1 more phosphorylated and basic. Collectively, these findings indicate that BZR1-mediated BR signaling pathways are actively induced for the first 1 or 2 d of dark incubation. To support this hypothesis, we tested whether the binding of BZR1 to a promoter of the *AIF2* gene is dependent on progression of the dark period using senescence-progressive leaves of *pBZR1::BZR1-EGFP* plants. *AIF2* contains at least two BZR1-binding *cis* motifs, *AIF2A* and *AIF2B*, in its promoter region (Supplementary Fig. S3; Kim *et al.*, 2017). We found that BZR1 binding to *AIF2B* was greatly enhanced during the first 24 h, maintained higher binding activity until 48 h, and gradually diminished thereafter (Fig. 2E). Accordingly, the level of AIF2 protein, determined based on GUS activity, decreased steadily as dark incubation progressed (Fig. 2F). Similar down-regulation of AIF2 during dark incubation was observed in another experiment using *pAIF2::AIF2-Luc* plants as a reporter (see Supplementary Fig. S4). Exogenous BL promoted leaf senescence, as determined by decreased amount of chlorophyll (Fig. 2G), and such promotion of BL-induced senescence was accompanied by a dramatic reduction of AIF2 protein accumulation in the leaves (Fig. 2H). ATSK21/BIN2 phosphorylates AIF2 to augment its protein stability (Kim *et al.*, 2017). We demonstrate that bikinin (BK, a BIN2 kinase inhibitor) treatment of the AIF2ox plants mimics BR effects by promoting senescence and corresponding reduction in AIF2 protein. Based on these observations, we concluded that BR-induced BIN2 inactivation, the consequently reduced accumulation of AIF2, and the aforementioned BZR1-mediated transcriptional repression contribute to dark-induced leaf senescence. It is unclear what causes down-regulation of AIF2 after 3 d of dark incubation when BZR1 transcription was decreased and its proteins were relatively phosphorylated. It is plausible that AIF2 is also regulated by other signaling pathways working at a later stage of dark-induced leaf senescence.

**Fig. 3.**
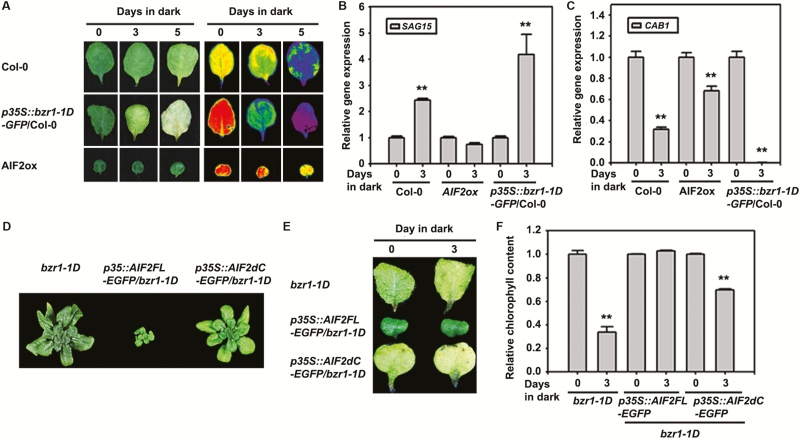
Genetic interaction of AIF2 with BZR1 in dark-triggered senescence. (A–C) Antagonistic effects of *bzr1-1D*-*GFP*-overexpressing *p35S::bzr1-1D-GFP*/Col-0 plants and full-length AIF2 protein-expressing AIF2ox (*p35S::AIF2FL-EGFP*/Col-0) on dark-triggered leaf senescence. The fourth leaves of 5-week-old plants were incubated in dark for different periods of time and their images/autofluorescence (A) or transcript accumulation of senescence-related *SAG15* (B) and *CAB1* (C) were examined using qRT-PCR. Transcript expression was normalized to that of the 0 d dark control of each plant, which was set to 1. (D–F) Epistatic effects of AIF2 over BZR1 on plant growth and dark-induced leaf senescence. Data show growth phenotypes of plants (D), dark-induced senescence phenotypes (E), and relative chlorophyll content of plants (F). Chlorophyll content was normalized to that of the 0 d dark control of each plant, which was set to 1. Bar graphs represent means ±SD, and asterisks on bars indicates a statistical difference from the 0 d sample of each plant: **P<0.01. (This figure is available in color at *JXB* online.)

**Fig. 4.**
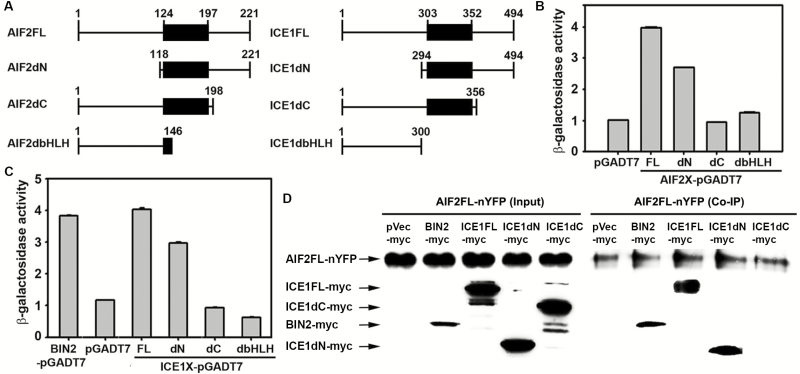
Identification of ICE1 as an AIF2 interactor. (A) Schematic diagram of different truncated forms of AIF2 and ICE1 used for generation of expression constructs described in Figs 3–6. The black box represents the basic helix–loop–helix (bHLH) region of AIF2 and ICE1, and the numbers indicate positions of amino acids. (B, C) *In vivo* interaction test of a full-length portion of ICE1 (ICE1FL) protein to diverse AIF2 proteins (B) or a full-length portion of AIF2 (AIF2FL) to ICE1 proteins (C). Level of interaction was quantified by measuring β-galactosidase activity as described in ‘Materials and methods’. BIN2-pGADT7 was used as a positive control for interaction with AIF2FL (Kim *et al.*, 2017). (D) *In planta* co-immunoprecipitation (Co-IP) assays of AIF2FL–nYFP protein with different myc-fused truncated versions of ICE1 proteins. Western blot analysis of the input fraction confirms expression of test proteins in tobacco.

To functionally evaluate these opposing regulatory roles of BZR1 and AIF2, we ectopically expressed the full-length coding region of AIF2 (*35S::AIF2FL-EGFP*) or C-terminal-deleted AIF2 (*35S::AIF2dC-EGFP*, deletion depicted in Fig. 4A) in a Col-0 or *bzr1-1D* genetic background. First, expression of a gain-of-function form of BZR1 (*35S::bzr1-1D*) in Col-0 plants further facilitated dark-induced leaf senescence as determined by decreasing leaf autofluorescence (Fig. 3A), increasing transcript level of *SAG15* (a marker of enhanced senescence) (Fig. 3B), and drastically reducing level of *CAB1*, a marker of active photosynthesis (Fig. 3C). In contrast, AIF2 overexpressing lines showed greatly retarded senescence phenotypes. Similarly, a senescence-promoting ethylene biosynthetic gene, *1-AMINOCYCLOPROPANE-1-CARBOXYLIC ACID SYNTHASE* (*ACS6*), and genes involved in ROS production and senescence, such as *CCX1* (a cation/Ca^2+^ exchanger gene; Li *et al.*, 2016) and *RbohF* (Koo *et al.*, 2017), were significantly increased in 3-day dark-incubated *35S::bzr1-1D-GFP* plants, while they were down-regulated in AIF2ox (see Supplementary Fig. S5). Moreover, ectopic expression of a full-length coding region of AIF2 (*35S::AIF2FL-EGFP* or AIF2ox) in *bzr1-1D* plants greatly antagonized the BR-enhanced growth-promoting phenotype of *bzr1-1D*, and deletion of the AIF2 C-terminus negated this AIF2-antagonized effect (Fig. 3D). Similarly, the accelerated dark-induced leaf senescence found in *bzr1-1D* plants was greatly suppressed by overexpression of AIF2FL–EGFP protein. This AIF2 effect was negated by C-terminus deletion (Fig. 3E, F). These results indicate that AIF2 is epistatic over BZR1 in regulation of plant growth and dark-induced leaf senescence. Thus, we concluded that BRs are biosynthetically activated when plants are subjected to dark, and BR-activated BZR1 transcriptionally down-regulates *AIF2* gene expression, resulting in promotion of dark-induced leaf senescence in Arabidopsis.

### AIF2 interactions with ICE1 *in vivo* are actively involved in transcriptional modulation of senescence-regulating genes to delay dark-induced leaf senescence

AIF2 is an atypical non-DNA-binding bHLH transcription factor and is a well-known member of the bHLH family that interacts with itself and with several other regulatory proteins. The bHLH transcription factors form complexes to activate or repress expression of genes that respond to diverse hormonal and developmental signals (Feller *et al.*, 2011). In general, DNA-binding bHLH proteins bind to the E-box and regulate cell-type and tissue-specific gene expression, whereas non-DNA-binding proteins bind to bHLH proteins and affect their DNA binding activity (Massari and Murre, 2000).

To further understand the molecular basis of AIF2 involvement in the retardation of leaf senescence, we screened 1.8×10^6^ yeast cells transformed by the Arabidopsis seedling cDNA library (Kim *et al.*, 2001) to identify potential AIF2-interacting proteins, using AIF2 as the bait in a yeast two-hybrid assay. Among the positive clones screened, 18 were overlapping cDNA clones encoding ICE1 (At3g26744), a nuclear-localized DNA-binding bHLH transcription factor shown to regulate freezing tolerance and cold acclimation (Chinnusamy *et al.*, 2003). Next, we generated deletion constructs of AIF2 and ICE1 and mapped their binding domains (deletion constructs depicted in Fig. 4A) by examining *in vivo* interactions in yeast cells. Interaction analysis revealed that both N- (188 amino acids) and C-termini (23 amino acids) of AIF2 are necessary for interaction with ICE1 (Fig. 4B). Specifically, co-transformation of C-terminal-deleted AIF2 (AIF2dC-pGADT7) with the full-length protein of ICE1 (ICE1FL-pGBKT7) abolished its interaction, implying that the C-terminus of AIF2 is the main ICE1-interacting domain, and the N-terminus may be needed to maintain proper conformation of the C-terminus to facilitate binding. The lack of interaction between ICE1FL and the C-terminal-deleted AIF2 (AIF2dC) was reconfirmed when transient co-expression of the ICE1FL–nYFP and the C-terminal portion of CFP-tagged AIF2dC (AIF2dC–cCFP) failed to yield a GFP signal in the nucleus of tobacco epidermal cells (see Supplementary Fig. S6A). Likewise, the C-terminal portion of ICE1 is required for its interaction with AIF2 in yeast and tobacco (Fig. 4C; Supplementary Fig. S6B). Additionally, AIF2FL co-immunoprecipitated with BIN2 (a positive control), ICE1FL, and the N-terminal-deleted ICE1 proteins (Fig. 4D). Consistent with our yeast two-hybrid and tobacco BiFC assays, deletion of the ICE1 C-terminus abolished its interaction with AIF2FL, confirming that this region is indispensable for its interaction with AIF2. Collectively, we demonstrated that ICE1 and AIF2 interact with each other through the respective regions of C-terminal domains that reside downstream of the bHLH domain.

**Fig. 5.**
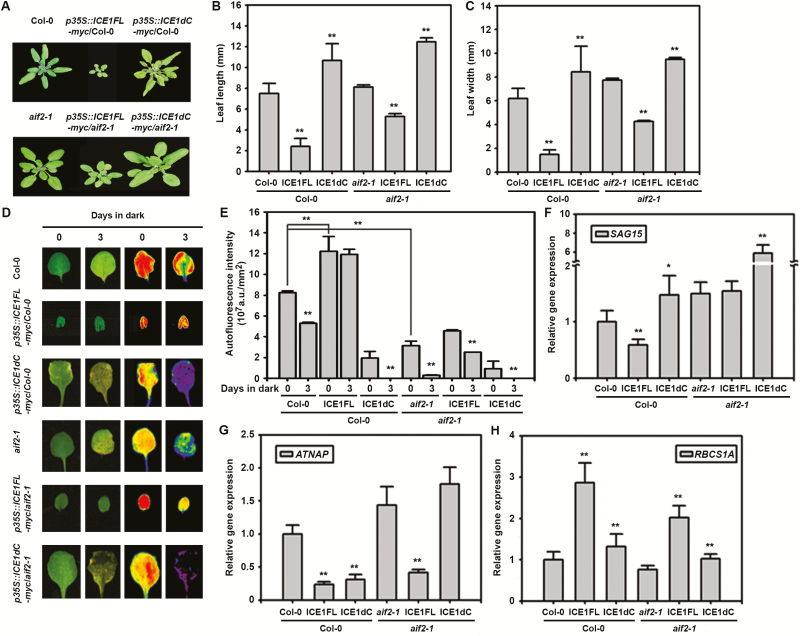
Functional analysis of AIF2–ICE1 interaction in regulation of senescence-related genes and retardation of dark-induced leaf senescence. (A–C) Full-length or C-terminus-deleted coding region of ICE1 was ectopically expressed in Col-0 or *aif2-1*. Data show aerial phenotypes (A) and the length (B) or width (C) of the fourth leaves of 5-week-old plants. (D, E) Dark-induced phenotypes of transgenic plants. The fourth leaves of plants described in (A) were subjected to dark for 3 d. Data show visible leaf phenotypes and autofluorescence (D) before and after the dark incubation and quantitative autofluorescence measurement (E). (F–H) Expression of senescence-related genes *SAG15* (F), *AtNAP* (G), and *RBCS1A* (H) in 0-day dark plants of Col-0 or *aif2-1*. Asterisks indicate differences between transgenic plants and non-transgenic Col-0 or *aif2-1* plants (B, C, F–H) or differences between mock- and dark-treated leaves (E) unless indicated by bracketed samples: *P<0.05, **P<0.01. (This figure is available in color at *JXB* online.)

We demonstrated that AIF2 is negatively involved in age- and dark-induced leaf senescence, and that AIF2 interacts *in planta* with ICE1 through its C-terminus. Thus, it is logical to evaluate if this interaction of AIF2 and ICE1 *in planta* is essential for retardation of dark-induced leaf senescence. To verify this, we first generated lines of transgenic plants that ectopically express the myc-tagged full-length coding region of ICE1 (*35S::ICE1FL-myc*) or C-terminus-deleted ICE1 (*35S::ICE1dC-myc*) in a Col-0 or *aif2-1* background (Fig. 5A). Ectopic expression of myc-tagged ICE1FL in Col-0 plants resulted in severe growth retardation, resulting in 70% reduction in length and 75% decrease in width of the leaves compared with those in Col-0 plants. In contrast, expression of the deleted form of ICE1, ICE1dC–myc, increased the leaf length and width by 40% and 42%, respectively (Fig. 5B, C). It seems that ectopically expressed ICE1dC–myc acts as a dominant negative factor replacing endogenous full-length AIF2, facilitating leaf growth. Similar regulation of leaf growth phenotype was found when ICE1FL or ICE1dC was introduced in the *aif2-1* loss-of-function mutant. Nonetheless, the degree of growth suppression was less severe, in that about 35% and 50% of the length and width were decreased, respectively. Consistent with the aforementioned ICE1FL-/ICE1dC-dependent modulation of growth phenotypes, a growth-inhibiting *IBH1* gene was transcriptionally up-regulated in ICE1FL-overexpressing plants, and deletion of the C-terminus, or expression of myc-tagged ICE1FL in an *aif2-1* background negated these effects (see Supplementary Fig. S7). In comparison, transcription of growth-promoting genes, such as *XTH4* and *EXPL2*, was counter-regulated compared with that of *IBH1*. These observations, together with the finding that AIF2 interacts *in vivo* with ICE1, suggest that AIF2 and ICE1 coordinately regulate leaf growth.

Next, we examined whether a coordinative action of AIF2 and ICE1 is necessary to effectively retard dark-induced leaf senescence. As expected, dark incubation of Col-0 plants led to significant leaf senescence progression as evidenced by a 30% reduction in autofluorescence gain after 3 d of incubation in the dark (Fig. 5D, E). Interestingly, ectopic expression of myc-tagged ICE1FL in Col-0 plants resulted in a greater than 50% increase in fluorescence gain and minimal loss of chloroplast after 3 d of incubation in the dark compared with that in Col-0 leaves. In contrast, expression of the C-terminus-deleted form of ICE1 showed an opposite regulation as seen for growth regulation. These observations imply that ICE1 is actively involved in maintenance of leaf photosynthesis and retardation of dark-induced senescence. The level of autofluorescence was significantly reduced in *aif2-1* plants compared with that of Col-0 plants, and dark incubation of *aif2-1* plants led to more effective leaf senescence, as evidenced by a greater than 90% reduction in autofluorescence gain. Transgenic expression of ICE1FL in *aif2-1* plants did not recover autofluorescence level to that of the Col-0 plants but did retard leaf senescence more effectively than *aif2-1* plants. Sequence alignment of AIF2 in Arabidopsis revealed that three BIN2-interacting AIF2 homologue proteins (AIF1, 3, and 4) shared the highly conserved helix–loop–helix domain and produced dwarfed plants when ectopically expressed in Arabidopsis (Wang *et al.*, 2009; Kim *et al.*, 2017). Therefore, it is plausible that these AIF2 homologues in *aif2-1* partly complemented AIF2 function to support ICE1FL-led retardation of leaf senescence. In comparison, deletion of the C-terminus of AIF2 and/or that of ICE1 efficiently abolished this senescence-retarding effect. Similar coordinative action of AIF2 and ICE1 for transcriptional regulation of senescence-related genes was found in genes that were up-regulated during senescence progression, such as *SAG15* (Fig. 5F) and *ATNAP* (Fig. 5G), and for the down-regulated gene, *RBCS1A* (Fig. 5H). Supporting these results, transient co-expression of AIF2FL and ICE1FL in tobacco leaves retarded the aging progress compared with that of an empty vector-transformed half of the leaf (see the first leaf in Supplementary Fig. S8A and compare the chlorophyll content in Supplementary Fig. S8B). Collectively, our findings reveal that proper interaction of AIF2 with ICE1 *in vivo* actively regulates this DNA-binding ICE1 transcription factor, and thus controls senescence-involved genes to efficiently delay dark-induced leaf senescence and retain green color.

### AIF2–ICE1 complex enhances ICE1 binding to the CBF2 promoter, resulting in transcriptional up-regulation, and inhibits ICE1 binding to the PIF4 promoter, leading to transcriptional down-regulation

**Fig. 6.**
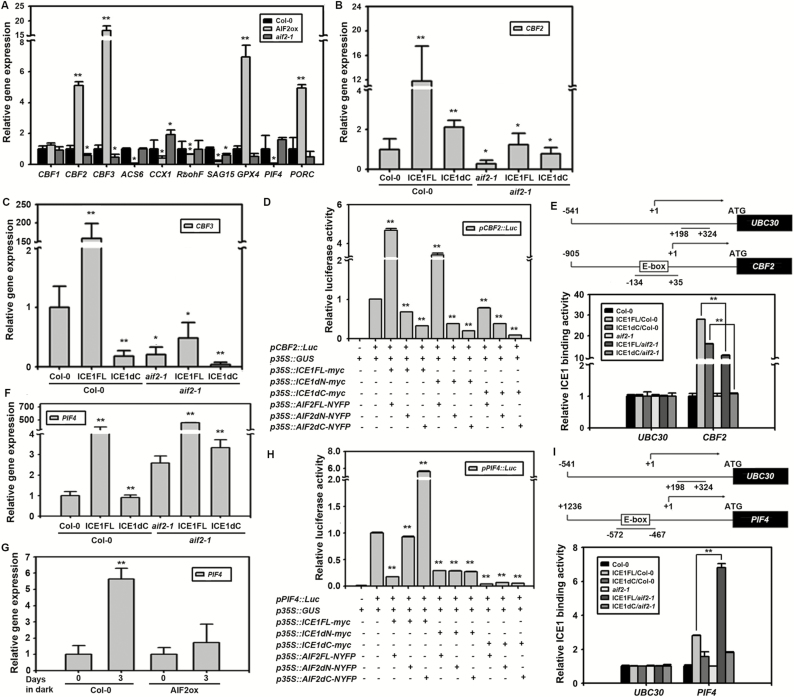
AIF2-dependent regulation of diverse senescence-related gene expression and promoter activities of *CBF2* and *PIF4* genes. (A) Gene expression analysis of *CBF*s and other senescence-related genes in Col-0, AIF2ox, and *aif2-1* plants. (B, C) AIF2–ICE1-dependent expression of *CBF2* (B) and *CBF3* (C) genes in ICE1FL- or ICE1dC-overexpressing Col-0 or *aif2-1* plants. (D) Transactivation test of *pCBF2::Luc* reporter by different effector combinations of ICE1 and AIF2 proteins. Experiments were performed as described in ‘Materials and methods’. (E) ChIP-qPCR analysis of ICE1Fl- or ICE1dC-binding activity to the *CBF2* gene promoter of plants described in (B). The promoter of *UBC30* gene served as a negative control for ICE1 binding activity. (F) AIF2–ICE1-dependent expression of *PIF4* gene in ICE1FL- or ICE1dC-overexpressing Col-0 or *aif2-1* plants. (G) Suppression of dark-induced *PIF4* up-regulation in AIF2 overexpressing plants. (H) Transactivation test of *pPIF4::Luc* reporter with different effector combinations of ICE1 and AIF2 proteins. (I) ChIP-qPCR analysis of ICE1FL- or ICE1dC-binding activity to the *PIF4* gene promoter of plants described in (B). Transcript accumulation of genes was normalized to that of Col-0 or AIF2ox plants before dark treatment (G) or to that of non-transgenic Col-0 plants (A–C, F), which was set to 1. ICE1 or ICE1dC binding to *CBF2* (E) or *PIF4* (I) promoters was normalized to that of either Col-0 plants or *aif2-1* mutants, which was set to 1. Asterisks on bars indicate differences from Col-0 plants (A), non-transgenic Col-0 or *aif2-1* plants (B, C, F), 0 d sample of Col-0 or AIF2ox plants (G), or reporter control expressing *p35S*::*GUS* together with *pCBF2*::*Luc* (D) or *pPIF4*::*Luc* (H) unless indicated by bracketed samples: *P<0.05, **P<0.01.

The unified ICE1/ICE2–CBFs pathway acts as the main transcriptional feedback control in regulation of freezing tolerance during cold acclimation (Kim *et al.*, 2015). We therefore examined whether known senescence-regulating genes are subjected to AIF2–ICE1-mediated transcriptional regulation during the senescence process. First, we found that expression of *CBF2* and *CBF3* was highly up-regulated in senescence-retarded AIF2ox leaves, with expression levels 5–15 times greater than that seen in Col-0, while these levels were down-regulated in *aif2-1* plants (Fig. 6A). In addition, transcript accumulation of the ethylene biosynthetic gene *ACS6* and the senescence-associated gene *SAG15* was significantly suppressed in the same AIF2ox plants. Similarly, expression of genes involved in ROS production and the subsequent senescence, *CCX1* and *RbohF*, was significantly reduced in AIF2ox plants. In contrast, expression of *GPX4* (a gene encoding glutathione peroxidase, which catalyses reduction of hydrogen peroxide) was drastically increased in the same AIF2ox plants. PIF4 promotes dark-induced ethylene biosynthesis to result in leaf senescence, and PIF4 directly binds to the promoter of *PROTOCHLOROPHYLLIDE OXIDOREDUCTASE C* (*PORC*) to negatively regulate *PORC* transcription and chloroplast activity (Song *et al.*, 2014; Toledo-Ortiz *et al.*, 2014). Consistent with the senescence-retarding role of AIF2, expression of *PIF4* was dramatically reduced in AIF2ox plants, while that of *PORC* was significantly up-regulated.

In this report, we demonstrated that expression of *RBCS1A*, a gene actively involved in photosynthesis, is coordinately regulated by AIF2 and ICE1 (Fig. 5H). As with the *RBCS1A* gene, expression of *CBF2* and *CBF3* was highly promoted by ectopic expression of ICE1FL, and deletion of the C-terminus dramatically reduced this ICE1FL effect in Col-0 plants (Fig. 6B, C). Moreover, expression of ICE1FL or ICE1dC in *aif2-1* plants further attenuated the ICE1FL effect, implying that expression of *CBF2* and *CBF3* is positively regulated by coordinated interactions between ICE1 and AIF2. A similar pattern of regulation was observed for *CBF1* and the senescence-promoting ethylene biosynthesis gene *ACS6* (see Supplementary Fig. S9). AIF2-dependent ICE1 regulation of *CBF2* expression was further confirmed by showing that co-expression of full-length AIF2 (*p35S::AIF2FL-EGFP*) and ICE1 (*p35S::ICE1FL-EGFP*) in tobacco leaves greatly enhanced *pCBF2-Luc* reporter expression (approximately 5-fold higher than the non-transformed mock control; Fig. 6D). In contrast, deletion of the C-terminus and/or the N-terminus of AIF2 and/or ICE1 severely attenuated this transcriptional enhancing effect and even suppressed reporter expression below the level of the non-transformed mock control. It is plausible that a deleted form of either ICE1 and/or AIF2 protein ectopically expressed in the system interferes with transcriptional regulation activities of their native tobacco homologs in a dominant-negative manner. ICE1/2 binds to MYC-recognition sequences (E-box) in *CBF* promoters and enhances their expression (Kim *et al.*, 2015). Our ChIP-PCR analysis confirmed that ICE1FL expressed in Col-0 plants directly bound to the E-box motif residing in the promoter of *CBF2*, and deletion of the C-terminus resulted in significantly reduced binding (Fig. 6E). Moreover, binding of ICE1FL to the *CBF2* promoter was further decreased when the protein was expressed in *aif2-1* plants, implying that AIF2 is a positive regulator of ICE1 transcription factor binding to the *CBF2* promoter and enhanced *CBF2* expression. Collectively, our data imply that AIF2 binds to ICE1, and this promotes expression of *CBF2* by directly binding to its promoter and delays dark-induced leaf senescence.

PIF4 promotes dark-induced ethylene biosynthesis and leaf senescence (Song *et al.*, 2014; Toledo-Ortiz *et al.*, 2014). Accordingly, *PIF4* expression was significantly down-regulated in AIF2ox plants (Fig. 6A). Surprisingly, ectopic expression of ICE1FL in Col-0 plants dramatically increased *PIF4* transcript expression (>250-fold compared with that in Col-0 plants), while deletion of the C-terminus nearly abolished this effect (Fig. 6F). The ICE1FL-induced *PIF4* promotion was more evident in *aif2-1* plants (>400-fold that of Col-0 plants). Thus, AIF2 may act as a negative regulator of ICE1 transcriptional activator for *PIF4* gene expression. Supporting this idea, ectopic expression of AIF2 significantly decreased the dark-induced *PIF4* gene expression compared with that of Col-0 plants (Fig. 6G). In addition, our tobacco transfection-based transient expression assay using *pPIF4-Luc* reporter revealed that *PIF4* gene expression was negatively controlled in an AIF2–ICE1 interaction-dependent manner (Fig. 6H). *PIF4* promoter-driven luciferase activity directed by ICE1FL was effectively suppressed when AIF2FL protein was co-expressed, while it was increased significantly when the C-terminus-deleted form of AIF2 was co-expressed with ICE1FL in tobacco. We analysed the promoter sequence of *PIF4* and identified a functional E-box motif to which ICE1 could bind (Fig. 6I). Interestingly, our ChIP-PCR analysis revealed that binding of ICE1FL to the E-box motif of *PIF4* promoter was significantly enhanced when ICE1 protein was expressed in *aif2-1* plants compared with its expression in Col-0 plants. Collectively, our data demonstrate that AIF2 together with ICE1 enhances *CBF2* expression and simultaneously suppresses expression of *PIF4* by oppositely modulating ICE1 binding to their promoters.

### Antagonistic down- and up-regulation of senescence-related genes through CBF2 and PIF4/WRKY/ANAC transcription factors explains AIF2–ICE1-mediated retardation of leaf senescence

**Fig. 7.**
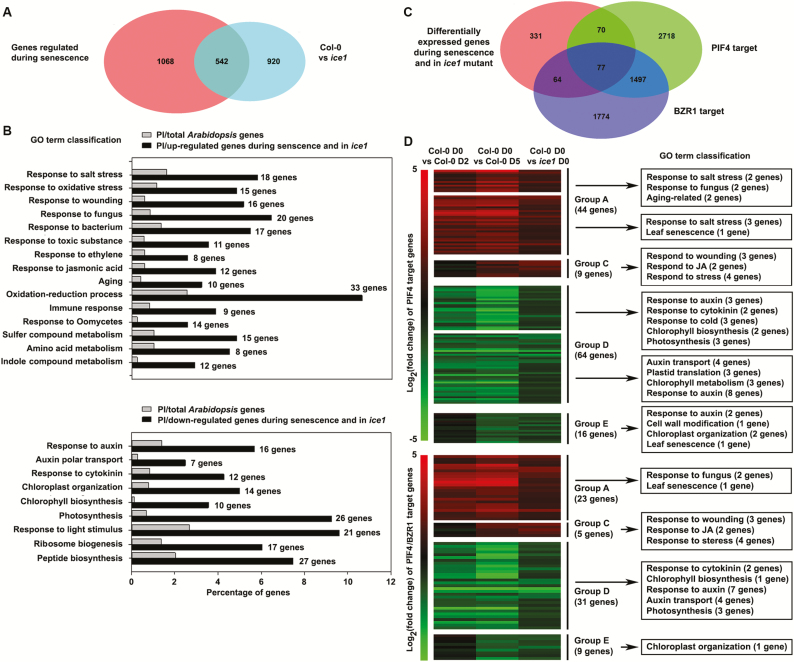
Transcriptome analysis of ICE1- and senescence-regulated genes. (A) Venn diagram showing overlapped differentially expressed genes (DEGs) in senescing leaves and *ice1* mutant. (B) GO categories significantly enriched with false discovery rate *P*-value<0.05 were identified in up- or down-regulated genes from senescing leaves and in *ice1* mutant. PI, percentage of genes belonging to the indicated GO category. (C) Venn diagram showing significant overlaps among ICE1- and senescence-regulated genes and PIF4-/BZR1-binding target genes. (D) Heatmaps showing PIF4- or PIF4- and BZR1-targeted genes in 542 ICE1- and senescence-regulated DEGs. Genes were classified into five groups (A to E) depending on regulation modes and were assigned with functional GO term classifications. Group A: up-regulated in Col-0 D2 and the same or further increased at D5. Group B: up-regulated in Col-0 D2 and decreased at D5. Group C: no significant change in Col-0 D2 and up-regulated at D5. Group D: down-regulated in Col-0 D2 and the same or further decreased at D5. Group E: no significant change in Col-0 D2 and down-regulated at D5. Up-regulation: log_2_(fold change)>1; no change: −1<log_2_(fold change)<1; down-regulation: log_2_(FC)<−1. (This figure is available in color at *JXB* online.)

ICE1 is a MYC-like bHLH transcription factor that binds to MYC-recognition motifs (Chinnusamy *et al.*, 2003; Kim *et al.*, 2015). We showed that ICE1 directly binds to *CBF2* and *PIF4* in an AIF2-dependent manner and thus negatively controls the progression of dark-induced leaf senescence. To further explore the involvement of ICE1 in regulation of leaf senescence, we retrieved publicly available transcriptome profile data to compare the leaves of Col-0 plants and those of *ice1* plants (Lee *et al.*, 2005) and subsequently classified them into five groups based on overlapping and differentially regulated genes in the leaves of Col-0 plants before (Col-0 D0) and 2 or 5 d after dark induction (Col-0 D2 and D5) (van der Graaff *et al.*, 2006) (groups A–E: see Fig. 7D legend). We found that 542 overlapping genes were differentially regulated both in senescing leaves and in *ice1-1* plants (Fig. 7A). Based on our results demonstrating retarded senescence in ICE1FL-overexpressing plants and enhanced senescence in ICE1dC-overexpressing plants, we expected a group of genes with a regulatory pattern similar to that in *ice1* and senescing Col-0, i.e. either up-regulated in both plants (total of 230 genes: genes in group A, B, C plus three genes not classified; Supplementary Fig. S10) or down-regulated in both plants (total of 279 genes: group D and E plus six genes not classified; Supplementary Fig. S10) as potential candidates of ICE1-regulated senescence genes (see Supplementary Table S4 for a list of genes). Functional classification based on Gene Ontology categories revealed that genes responding to senescence-promoting hormones (JA, ethylene) as well as genes responding to salt, oxidative stress, wounding, bacterial/fungal infection, and toxins were significantly enriched for up-regulation both in senescing leaves and in *ice1-1* plants (Fig. 7B). In addition, genes involved in compound metabolism such as those for sulfur, amino acids, and indole were up-regulated both in aging leaves and in *ice1* plants. In contrast, genes related to maintaining growing stages (auxin response/transport, cytokinin response, photosynthesis, etc.) were significantly down-regulated both in Col-0 plants undergoing senescence and in *ice1* plants. These results suggest that ICE1-induced retardation of leaf senescence involves suppression of diverse senescence-promoting hormones and stress responses, as well as promotion of auxin/cytokinin and other diverse cellular activities such as photosynthesis and cell expansion.

We showed that ICE1 and AIF2 coordinately enhance the expression of *CBF2*, while suppressing the expression of *PIF4* by modulating ICE1 binding to their promoters (Fig. 6). *CBF1*, *CBF2*, and *CBF3* encode closely related AP2/ERF DNA-binding proteins that recognize the C-repeat (CRT)/dehydration-responsive element (DRE) present in the promoters of CBF-regulated genes (Park *et al.*, 2015). Thus, it is plausible that ICE1-regulated senescence genes include *CBF*-regulated genes. For example, genes for protochlorophyllide oxidoreductase B (*PORB*, At4g27440) and PORC (At1g03630), key enzymes for chlorophyll biosynthesis, were significantly down-regulated in senescing leaves and in *ice1* mutants (see Supplementary Table S4) while they were highly up-regulated in *CBF2*-overexpressing plants (Sharabi-Schwager *et al.*, 2010). PIFs are also actively involved in facilitation of age-triggered and dark-induced senescence by promoting ethylene/ABA biosynthesis and signaling pathways (Song *et al.*, 2014; Sakuraba *et al.*, 2014). Of the 542 senescence- and *ice1*-regulated genes, 147 were directly regulated by PIF4 binding to their promoters (Fig. 7C). In particular, a group of genes involved in maintaining plants’ green status, such as auxin response/transport, cytokinin response, and chlorophyll biosynthesis and photosynthesis, were overrepresented in PIF4-bound and repressed genes (Fig. 7D). GOLDEN TWO-LIKE 2 (GLK2) and GLK1 transcription factors promote the expression of genes required for chlorophyll biosynthesis, light harvesting, and electron transport functions (Waters *et al.*, 2009). Interestingly, PIF4 directly bound to the promoter of *GLK2*, a gene that is repressed during senescence (Supplementary Table S5; Oh *et al.*, 2012). Recent functional analyses show that Arabidopsis WRKY and NAC transcription factors play important roles in facilitating leaf senescence (Koyama, 2014). We also found that non-PIF4 targets such as *WRKY 6*, *WRKY25*, *WRKY26*, *WRKY45*, and *WRKY75* and those of *ANAC021* (*AtNAC1*), *ANAC047*, and *ANAC087* were up-regulated both in senescing leaves and in *ice1* plants (Supplementary Table S5). BZR1 and PIF4 interact with each other *in vitro* and *in vivo* and synergistically regulate many of these target genes (Oh *et al.*, 2012). Of 542 senescence- and *ice1*-regulated genes, 77 were directly regulated by both PIF4 and BZR1 binding to their promoters, and many of these were related to photosynthesis and response to auxin and cytokinin (Fig. 7C, D).

## Discussion

Leaf senescence is crucial for plant survival as it is linked to efficient nutrient and energy recycling, resulting in massive changes in plant metabolism. Being a key process, senescence is tightly controlled by recruiting transcription factors as switches to regulate gene expression (Woo *et al.*, 2013). PIF4 and PIF5 are major transcription factors facilitating the onset of dark-induced senescence, mainly via positive actions on the biosynthesis and signaling pathways of two senescence-promoting hormones, ethylene and ABA (Sakuraba *et al.*, 2014; Song *et al.*, 2014; Liebsch and Keech, 2016). Interestingly, PIF4 and PIF5 bind to the promoter region of key BR biosynthetic genes, such as *DWF4* and *BR6ox2*, to directly promote their expression (Wei *et al.*, 2017). BES1 homodimers bind to conserved BRRE- and G-box elements in BR biosynthetic promoters and inhibit their expression during the day, while an elevated level of PIF4 at dawn competes for BES1 homodimer formation, resulting in de-repressed BR biosynthesis and an increased BR level (Martínez *et al.*, 2018). A shade or dark condition, which has a low red: far-red ratio, inhibits phyB-mediated PIF degradation, thus forming multiple coherent feed-forward loops with bZIP (ABI5 and EEL), EIN3, and ORE1 to induce dark-induced leaf senescence (Sakuraba *et al.*, 2014; Liebsch and Keech, 2016). Additionally, BZR1 and PIF4 interact with each other *in vitro* and *in vivo*, as they bind to nearly 2000 common target genes and synergistically regulate many of these target genes in response to BR, darkness, or heat (Oh *et al.*, 2012).

**Fig. 8.**
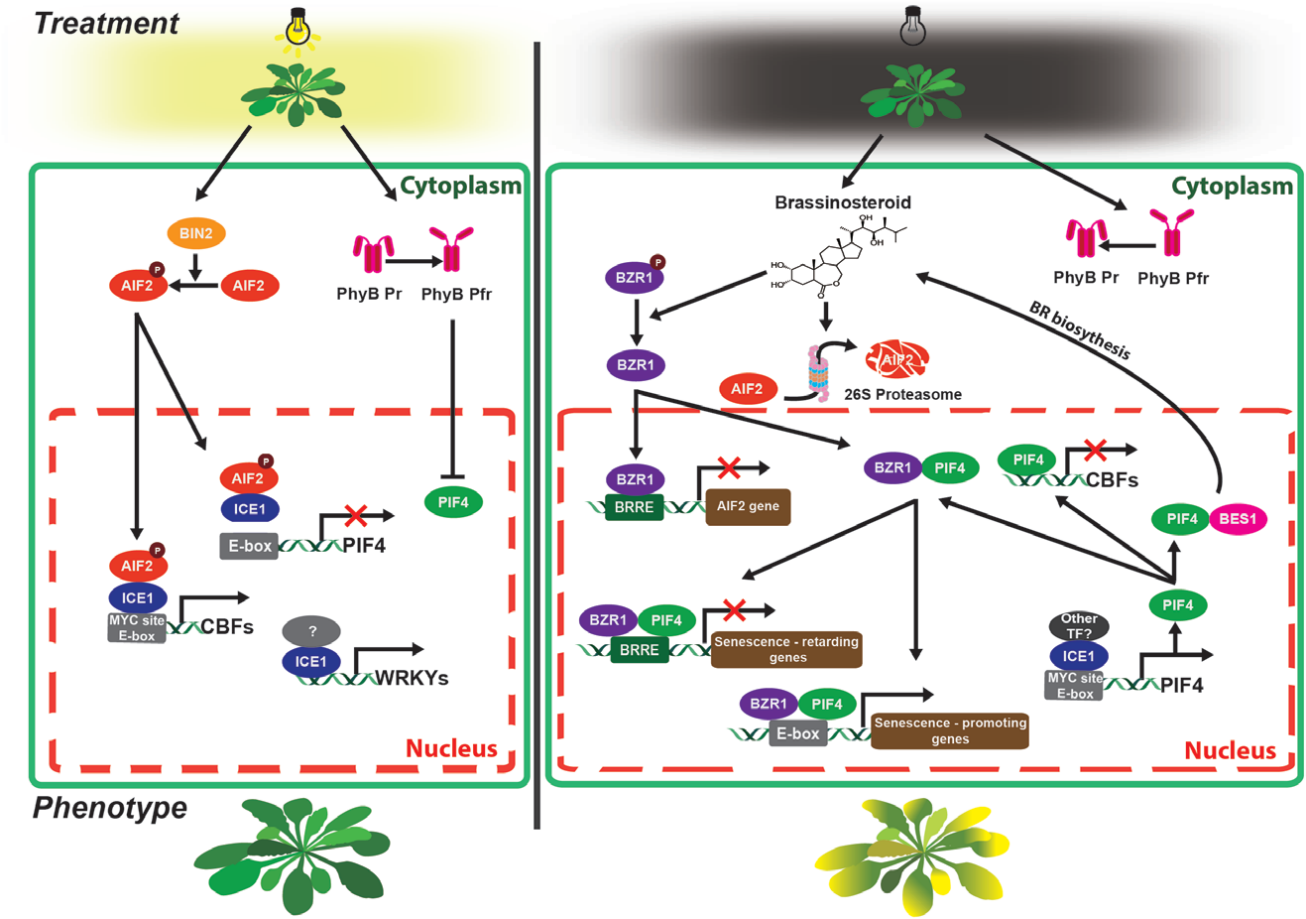
A proposed model of AIF2–ICE1-mediated retardation of dark-induced senescence. Light on growing and maturing leaves leads to degradation of senescence-promoting PIF4 transcription factors through phyB-mediated repression. At the same time, BIN2-activated AIF2 binds to ICE1 to directly down-regulate *PIF4* expression and antagonistically up-regulate *CBF*, which promote senescence-retarding genes such as those involved in auxin biosynthesis/transport and chlorophyll biosynthesis/chloroplast organization. Senescence-promoting *WRKYs* are also subject to down-regulation by ICE1. As plants are subjected to darkness, the early senescence-responding *PIF4* gene, together with the dark-driven stabilization of PIF4, promotes BR biosynthesis and BZR1 activation, leading to degradation of AIF2. As dark incubation progresses, an exponential increase of PIF4 suppresses senescence-retarding genes such as *CBF* and facilitates expression of senescence-promoting gene, such as those involved in ethylene/JA biosynthesis and their signaling pathways. **→**, promotion; ┤, suppression; BRRE, BR response element; TF, transcription factor. (This figure is available in color at *JXB* online.)

In this study, we provided molecular evidence supporting the important retardation role of the AIF2-ICE1-mediated negative BR signaling pathway in age- and dark-induced leaf senescence, achieved via enhanced gene expression for senescence-retarding *CBF*s and antagonistic down-regulation of senescence-promoting *PIF4* (Fig. 8, a model explaining AIF2–ICE1-mediated retardation of dark-induced senescence). In contrast, a high level of brassinosteroid or *bzr1-1D* plants (a gain-of-function mutant of the BR positive signaling pathway) enhanced leaf senescence through transcriptional and post-translational suppression of AIF2 and up-regulation of *PIF4* during senescence progression (Figs 2, 3). It is highly probable that an artificially induced dark condition relieves light-driven and phyB-mediated PIF4 repression, and this PIF4 subsequently promotes BR biosynthesis and the BZR1-mediated BR signaling pathway leading to suppression of AIF2. Thus, BR-induced BZR1 activation, together with AIF2 suppression might provide positive feedback to accelerate the early stage of dark-induced leaf senescence by enhancing BR biosynthesis and *PIF4* gene expression, which then promotes ethylene/ABA biosynthesis/signaling pathways. We showed that *CBF*s are highly up-regulated in senescence-retarding AIF2ox plants, while *PIF4* is down-regulated in an AIF2–ICE1-dependent manner (Fig. 6). In fact, PIF3/4/7 and EIN3 bind to *CBF* promoters and repress their expression during cold stress, long-day periods, and in the ethylene-induced signaling pathway (Lee and Thomashow, 2012; Shi *et al.*, 2012; Jiang *et al.*, 2017). Therefore, it is plausible that a decrease in AIF2 activity, together with dark-triggered accumulation of PIF4 leading to down-regulation of *CBFs*, resulted in acceleration of leaf senescence. Supporting our model, ectopic expression of ICE1 in Arabidopsis delayed ethylene- and stress-induced leaf senescence (Budhagatapalli *et al.*, 2016). Moreover, overexpression of *CBF2* and *CBF3* delayed not only dark-induced leaf senescence, but also the artificial leaf senescence triggered by exogenously supplied ethylene, ABA, SA, and JA (Sharabi-Schwager *et al.*, 2010; Hu *et al.*, 2017). Here, we showed that AIF2 acts as a transcriptional activator of *CBF2* and *CBF3* (by interacting with ICE1), while it functions as a transcriptional repressor of the senescence-promoting *PIF4* gene (Figs 4, 6). Similarly, ICE1/2 and CAMTA1–3 bind to the MYC-recognition sequences in *CBF* promoters and enhance their expression (Doherty *et al.*, 2009). In contrast, JAZ1 and JAZ4, which are repressors of JA signaling, physically interact with ICE1 and ICE2 to repress their transcription activity, thereby negatively modulating *CBF* expression (Hu *et al.*, 2013). In the future, it would be valuable to identify the ICE1-bound positive regulator of *PIF* expression acting contrary to AIF2 in dark.

WRKYs and NACs are also ICE1-regulated transcription factors and are highly up-regulated in senescing leaves, although not directly by PIF4 regulation (see Supplementary Table S5). REPRESSOR of *ga1-3* (RGA; a negative regulator of gibberellin signaling) and RGA-LIKE 1 (RGL1) interact with WRKY6 and WRKY45, respectively, and negatively regulate up-regulation of senescence-related gene expression to retard both dark-induced (Zhang *et al.*, 2018) and age-triggered leaf senescence (Chen *et al.*, 2017). Transcription of *WRKY75* was repressed by cytokinin and induced by age, SA, ethylene, JA, and ABA. In turn, WRKY75 promoted SA biosynthesis by directly activating *SA*-*INDUCTION-DEFICIENT 2* (*SID2*) transcription, forming an amplification loop to accelerate age-dependent leaf senescence (Guo *et al.*, 2017). Thus, it appears that at least 542 ICE1-regulated and senescence-related genes (Fig. 7) are coordinately regulated, in part, by the dark-induced transcription activities of PIF4, WRKYs, and CBFs on senescence-promoting and/or -inhibiting hormones and downstream genes.

Stress tolerance and life span shows tight correlation in plants, and stresses generally accelerate leaf senescence. Considering that AIF2 is also dramatically up-regulated in drought-stressed Arabidopsis (Ding *et al.*, 2013) and is functional in salt stress tolerance (Guan *et al.*, 2013), this study provides further evidence supporting the stress resistance theory of aging, which hypothesizes that increased resistance to intrinsic and extrinsic stresses leads to a prolonged life span.

## Supplementary data

Supplementary data are available at *JXB* online.

Fig. S1. Age-dependent progression of leaf senescence in Col-0, AIF2ox, and *aif2-1* plants.

Fig. S2. Effects of brassinosteroid and its biosynthesis- and signaling-related genetic backgrounds in dark-induced leaf senescence.

Fig. S3. Schematic diagram of PCR-amplified potential BZR1-binding sites (E-box and BRRE) found in promoters of test genes.

Fig. S4. Time-dependent AIF2–Luc expression activity in dark-triggered *pAIF2::AIF2-Luc* plants.

Fig. S5. Antagonistic effects of *bzr1-1D*-*GFP*-overexpressing *p35S::bzr1-1D-GFP*/Col-0 plants and the full-length AIF2 protein-expressing AIF2ox (*p35S::AIF2FL-EGFP*/Col-0) on dark-triggered leaf senescence.

Fig. S6. *In vivo* interaction test of AIF2 with ICE1 in tobacco.

Fig. S7. Functional analysis of AIF2–ICE1 interaction in the regulation of growth-related gene expression.

Fig. S8. Functional analysis of AIF2–ICE1 interaction in tobacco, leading to retardation of dark-induced leaf senescence.

Fig. S9. AIF2/ICE1-dependent expression of *CBF1* and *ACS6* genes in the ICE1FL- or ICE1dC-overexpressing Col-0 or *aif2-1* plants.

Fig. S10. Heatmap and gene ontology classification of PIF4- or non-PIF4-targeted genes in 542 ICE1- and senescence-regulated DEGs.

Table S1. Primers used in cDNA or promoter amplification of *AIF2*, *ICE1*, *BIN2*, *CBF2*, and *PIF4*.

Table S2. Primers used in quantitative real-time RT-PCR analysis.

Table S3. Primers used in ChIP-qPCR analysis.

Table S4. A list of differentially regulated genes grouped according to expression in Col-0 D0, D2, D5, and *ice1* mutant.

Table S5. Transcription factors differentially regulated both in leaves undergoing senescence and *ice1* mutant.

erz533_suppl_Supplementary_Figures_S1-S10

erz533_suppl_Supplementary_Table_S4

erz533_suppl_Supplementary_Table_S5

erz533_suppl_Supplementary_Tables_S1-S3

erz533_suppl_Supplementary_Data
