# Corrigendum: ATBS1-INTERACTING FACTOR 2 negatively regulates dark- and brassinosteroid-induced leaf senescence through interactions with INDUCER OF CBF EXPRESSION 1

**DOI:** 10.1093/jxb/eraa013

**Published:** 2020-02-12

**Authors:** Yoon Kim, Seon-U Park, Dong-Min Shin, Giang Pham, You Seung Jeong, Soo-Hwan Kim

**Affiliations:** Division of Biological Science and Technology, Yonsei University, Yonseidaegil, Wonju-Si, Republic of Korea

*Journal of Experimental Botany,* Advance Access publication: 30 November 2019, doi: 10.1093/jxb/erz533

In the above article, author Yoon Kim’s name was initially published as ‘Yoon Ki.’ This has now been corrected.

