# Supplementary material for: ATBS1-INTERACTING FACTOR 2 negatively regulates dark- and brassinosteroid-induced leaf senescence through interactions with INDUCER OF CBF EXPRESSION 1
Source: J Exp Bot. 2019 Nov 30;71(4):1475–90. doi: 10.1093/jxb/erz533 (PMC7031079; doi:10.1093/jxb/erz533)
Supplement: erz533_suppl_Supplementary_Figures_S1-S10 [file erz533_suppl_supplementary_figures_s1-s10.pdf]

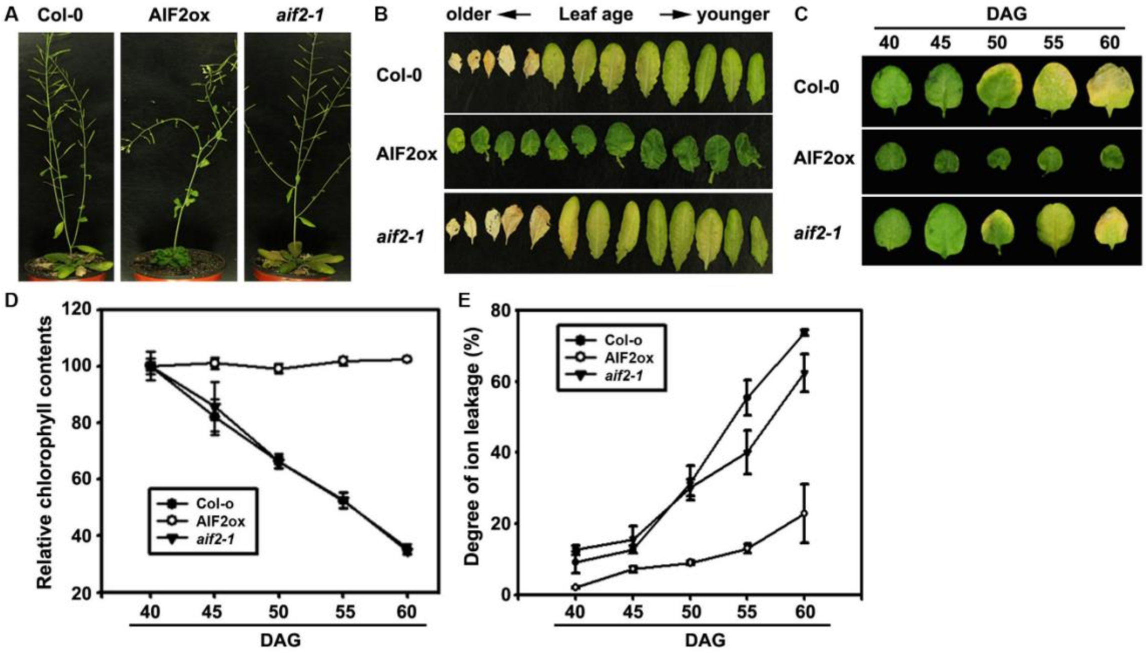

Fig. S1. Age-dependent progression of leaf senescence in Col-0, AIF2ox, and *aif2-1* plants. Typical age-dependent senescence phenotype of 10-week-old Col-0, AIF2ox, and *aif2-1* plants. Pictures show aerial view of plants (A) and development-dependent senescence phenotype of rosette leaves, displayed according to position along the stem (B). Age-dependent senescence phenotype of the fourth rosette leaf is displayed from 40 days after germination (40 DAG) to 60 DAG (C), and senescence progression was analyzed by measuring total chlorophyll (D) or ion leakage from the leaves (E). Chlorophyll contents of Col-0. AIF2ox, and *aif2-1* plants were normalized to that of 40 DAG plants (D), which was set to 100.

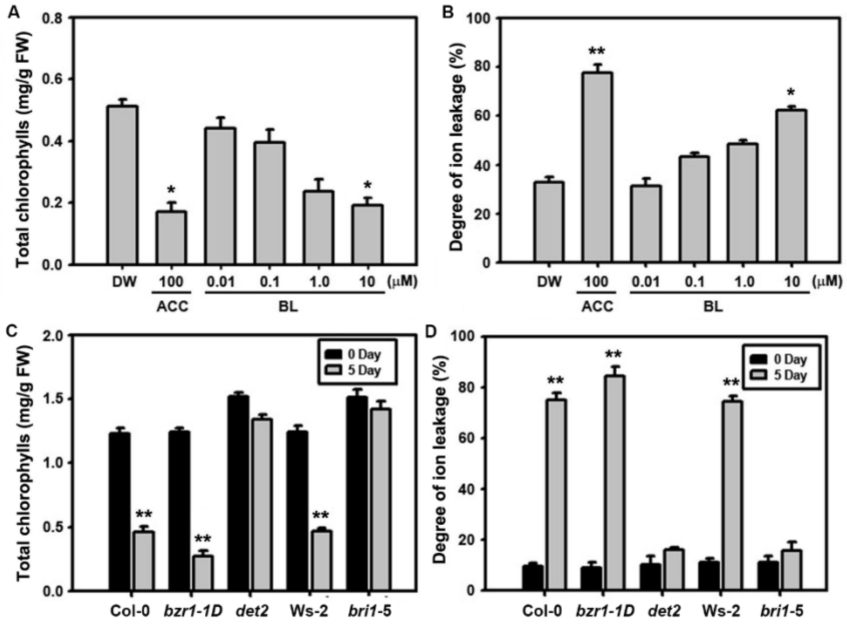

Fig. S2. Effects of brassinosteroid and its biosynthesis- and signaling-related genetic backgrounds in dark-induced leaf senescence. (A, B) Leaves of 5-week-old *Arabidopsi*s wild-type plants (Col-0) were incubated for 5 days in darkness in the presence or absence of various concentrations of brassinolide (BL), an active BR. ACC, a precursor of ethylene, was used as a positive control for senescence promotion. Senescence progression was analyzed by measuring total chlorophyll (A) or ion leakage from leaves (B). (C, D) Leaves of 5-week-old BR biosynthesis- or signaling-related mutants were incubated for 5 days in darkness, and senescence progression was analyzed by measuring total chlorophyll (C) or ion leakage from leaves (D). Bar graphs represent mean ± SD, and an asterisk (*) indicates statistical difference from either the mock-treated Col-0 control (A, B) or 0-day samples (C, D) at $P< 0.05$, and two asterisks (**) represents difference at $P< 0.01$.

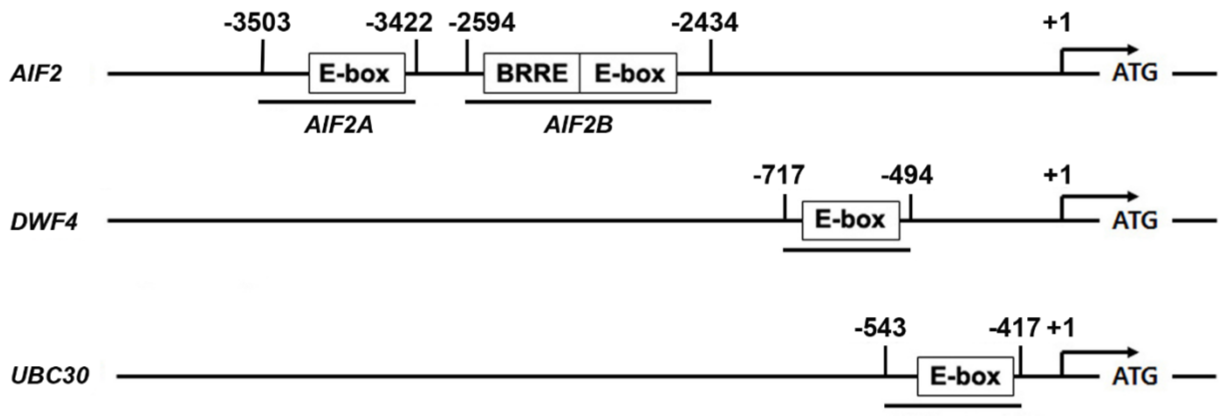

Fig. S3. Schematic diagram of PCR-amplified potential BZR1-binding sites (E-box and BRRE) found in promoters of test genes. The 5’ and 3’ positions of each fragment are indicated with numbers. +1 represents the transcriptional initiation site, and each negative number indicates the upstream position. Nucleotide positions and fragment lengths are not to scale.

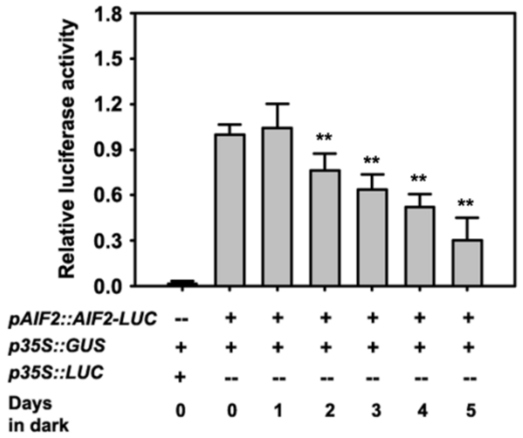

Fig. S4. Time-dependent AIF2-Luc expression activity in dark-triggered *pAIF2::AIF2-Luc* plants. Leaves of plants were subjected to different durations of dark, and luciferase activity was measured as described in the Materials and Methods. Luciferase activities were normalized to that of the 0-day dark control of *pAIF2::AIF2-Luc* plants which was set to 1. Bar graphs represent mean ± SD, and two asterisks (**) on bars indicates a statistical difference from the 0-day dark sample of *pAIF2::AIF2-Luc* plants at $P< 0.01$.

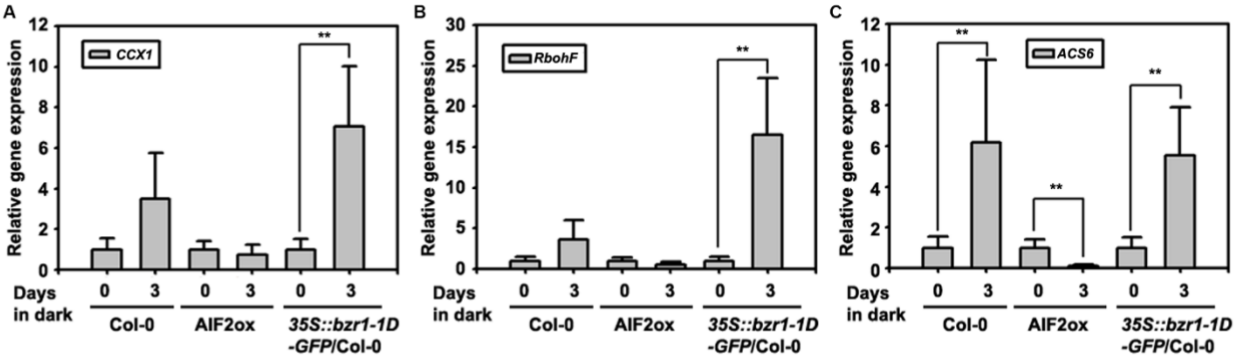

Fig. S5. Antagonistic effects of *bzr1-1D-GFP*-overexpressing *p35S::bzr1-1D-GFP*/Col-0 plants and the full-length AIF2 protein-expressing AIF2ox (*p35S::AIF2FL-EGFP*/Col-0) on dark-triggered leaf senescence. (A-C) The fourth leaves of 5-week-old plants were incubated in dark for different periods of time, and transcript accumulation of senescence-related *CCX1* (A), *RbohF* (B), and *ACS6* (C) was examined using qRT-PCR. Transcript expression was normalized to that of the 0-day dark control of each plant, which was set to 1. Two asterisks (**) on bracketed samples represents a statistical difference between the two compared samples at P< 0.01.

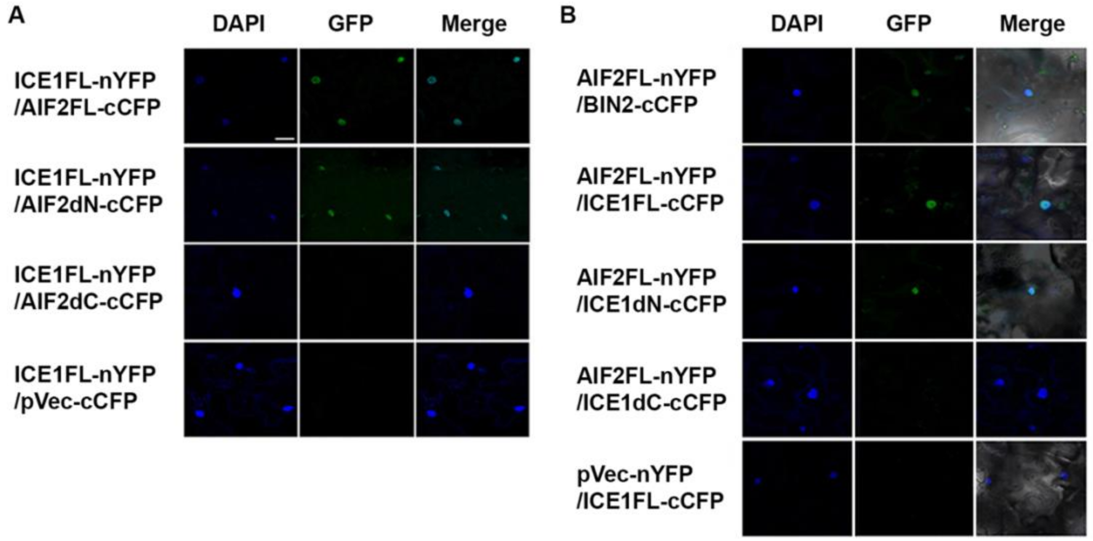

Fig. S6. *In vivo* interaction test of AIF2 with ICE1 in tobacco. To test protein interactions *in planta* by BiFC, cDNAs encoding either full-length or truncated forms of *AIF2*, *ICE1*, and *BIN2* (Fig. 4A) were PCR-amplified (Table S1) and cloned in Gateway-compatible binary vectors, *pPZP312-nYFP* or *pPZP312-cCFP*, in fusion with the N-terminus (nYFP) or the C-terminus of CFP (cYFP), respectively. Next, a combination of *Agrobacterium tumefaciens* GV301 containing the full-length or partial coding regions of *AIF2*, *ICE1*, and *BIN2* (*p35S::ICE1FL-nYFP*, *p35S::AIF2FL-cCFP*, *AIF2dN-cCFP*, *p35S::AIF2dC-cCFP*, *pVec-cCFP*, *p35S::AIF2FL-nYFP*, *p35S::BIN2-cCFP*, *p35S::ICE1FL-cCFP*, *p35S::ICE1dC-cCFP*, and *pVec-nYFP*) was co-infiltrated through the underside of 4-week-old tobacco leaves. After 36-48 h of incubation, epidermal cell layers were examined for fluorescence using a Zeiss LSM 710 confocal laser scanning microscope. This image shows GFP epifluorescence of epidermal cells, indicating interaction between the test proteins. DAPI staining localizes the position of nucleus in the cell. Bar indicates 20 μm in length. BIN2-cCFP was used as a positive control for interaction with AIF2FL.

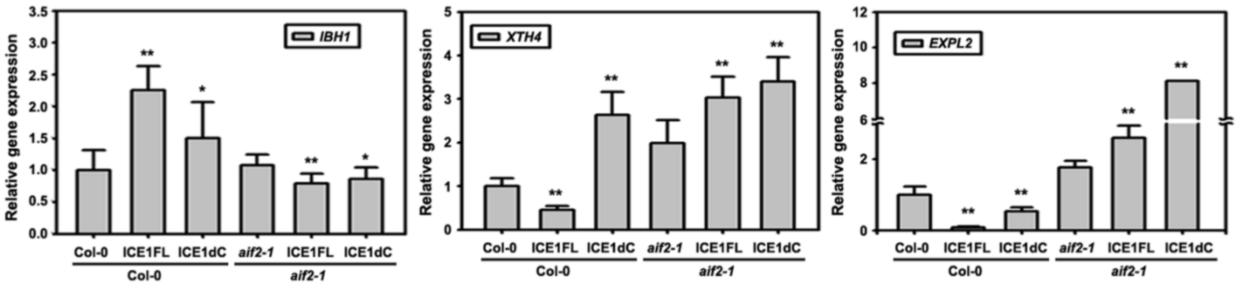

Fig. S7. Functional analysis of AIF2-ICE1 interaction in the regulation of growth-related gene expression. The full-length or C-terminus-deleted coding region of ICE1 was ectopically-expressed in Col-0 or *aif2-1*. Data show relative transcript expression of *IBH1*, *XTH4*, and *EXPL2* genes measured from the fourth leaves of 5-week-old plants. Statistical differences between the transgenic and non-transgenic Col-0 or *aif2-1* plants are noted with asterisks.

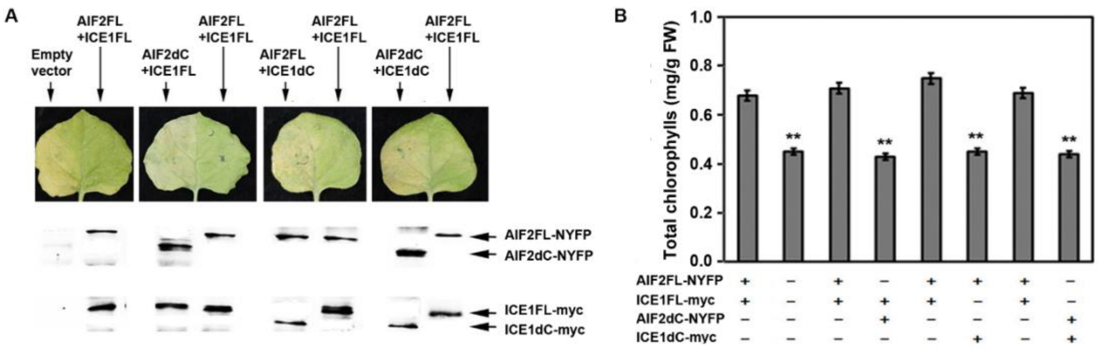

Fig. S8. Functional analysis of AIF2-ICE1 interaction in tobacco, leading to retardation of dark-induced leaf senescence. Different combinations of nYFP-fused AIF2 and MYC-tagged ICE1 proteins were co-expressed in tobacco for 3 days in dark to examine their effects on dark-triggered leaf senescence. Progression of senescence was visible in (A) and was analyzed by measuring total chlorophyll contents in the infiltrated leaf area (B). Western blot analysis confirms expression of test proteins in tobacco.

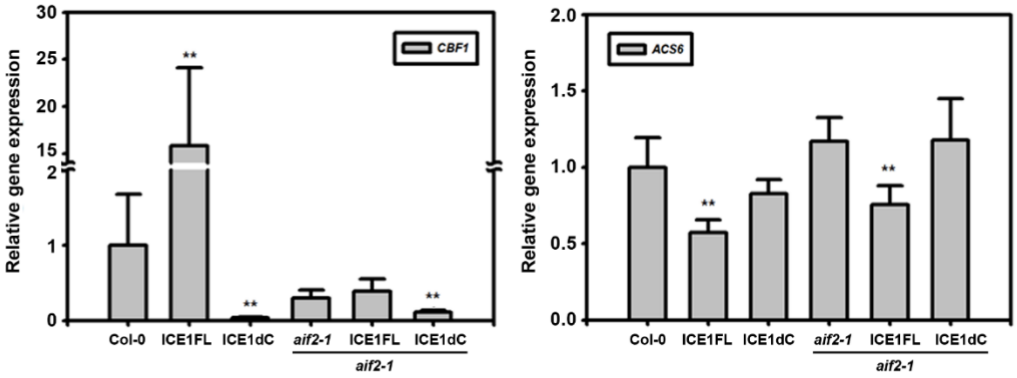

Fig. S9. AIF2/ICE1-dependent expression of *CBF1* and *ACS6* genes in the ICE1FL- or ICE1dC-over-expressing Col-0 or *aif2-1* plants. The fourth leaves of 5-week-old plants were used for measuring the transcript levels using qRT-PCR. Transcript levels of genes were normalized to those of non-transgenic Col-0 plants, which were set to 1. Statistical differences between the transgenic and non-transgenic Col-0 or *aif2-1* plants are noted with asterisks.

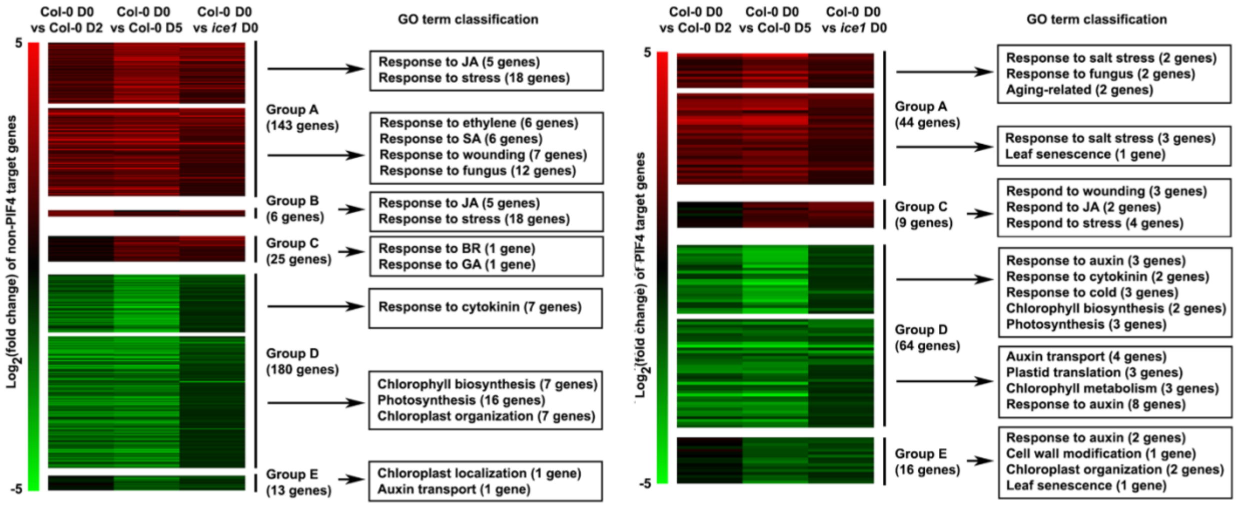

Fig. S10. Heatmap and gene ontology classification of PIF4- or non-PIF4-targeted genes in 542 ICE1- and senescence-regulated DEGs. Genes were classified into five groups (A to E) depending on regulation modes and were assigned with functional GO term classifications. Group A: upregulated in Col-0 D2 and the same or further increased at D5. Group B: Upregulated in Col-0 D2 and decreased at D5. Group C: no significant changes in Col-0 D2 and upregulated at D5. Group D: downregulated in Col-0 D2 and the same or further decreased at D5. Group E: no significant change in Col-0 D2 and downregulated at D5. Upregulation: $\log_2$ (FC) >1. No change: -1 <$\log_2$ (FC) <1. Downregulation: $\log_2$ (FC) <-1. FC: fold change.
