# Supplementary material for: ATBS1-INTERACTING FACTOR 2 negatively regulates dark- and brassinosteroid-induced leaf senescence through interactions with INDUCER OF CBF EXPRESSION 1
Source: J Exp Bot. 2019 Nov 30;71(4):1475–90. doi: 10.1093/jxb/erz533 (PMC7031079; doi:10.1093/jxb/erz533)
Supplement: erz533_suppl_Supplementary_Table_S5 [file erz533_suppl_supplementary_table_s5.pdf]

**Table S5.** Transcription factors differentially regulated both in leaves undergoing senescence and in *ice1* mutant						
	Locus taq	Gene symbol	Description	Expression pattern indicated in Figure 8C	Presence of PIF4-, or BZR1-binding motif	Senescence-related reference
Up-regulated	At2g35940	BLH1	BEL1-like homeodomain protein 1	A	PIF4, BZR1	
	At1g05710		Basic helix-loop-helix DNA-binding superfamily protein	A	PIF4, BZR1	
	At2g42280	AKS3	Basic helix-loop-helix DNA-binding superfamily protein	A	PIF4, BZR1	
	At1g62300	AtWRKY6	Probable WRKY transcription factor 6	A	-	[1]Lim et al., 2018; [2]Zhang et al., 2018
	At2g30250	AtWRKY25	Probable WRKY transcription factor 25	A	-	
	At5g07100	AtWRKY26	Probable WRKY transcription factor 26	A	-	

	At3g01970	AtWRKY45	Probable WRKY transcription factor 45	A	-	[3]Chen et al., 2017
	At5g13080	AtWRKY75	WRKY75	A	-	[4]Guo et al., 2017
	At1g56010	ANAC021 (AtNAC1)	NAC domain-containing protein 21/22	A	-	
	At3g04070	ANAC047 (SHYG)	NAC transcription factor 47; SPEEDY HYPONASTIC GROWTH	A	-	[5]Kim et al., 2014
	At5g18270	ANAC087	NAC domain-containing protein 87	A	-	[6]Huysmans et al., 2018
	At5g13330	Rap2.6L	ERF subfamily B-4 of ERF/AP2 transcription factor	A	-	
	At4g36990	AT-HSFB1	Heat stress transcription factor B-1	A	-	
	At4g17500	AtERF1	Ethylene-responsive transcription factor 1A	C	-	[7]Kuang et al., 2012
	At5g59820	AtZAT12	Zinc finger protein ZAT12	C	PIF4, BZR1	
Down-regulated	At2g18300	HBI1	Basic helix-loop-helix DNA-binding	D	PIF4, BZR1	

			superfamily protein			
	At2g25900	AtCTH (AtTZF1)	Zinc finger CCCH domain-containing protein 23	D	-	
	At5g15310	AtMIXTA	MYB16	D	-	
	At4g14540	NF-YB3	Nuclear transcription factor Y subunit B-3	D	-	
	At5g44190	AtGLK2	Transcription factor GLK2	E	PIF4	[8]Zubo et al., 2018; [9]Song et al., 2014
	At1g71030	AtMYBL2	Putative MYB transcription factor	E	-	
	At2g39250	SNZ	AP2-like ethylene-responsive transcription factor SNZ			

[1]**Lim J, Park J-H, Jung S, Hwang D, Nam HG, Hong S. 2018.** Antagonistic roles of PhyA and PhyB in far-red light-dependent leaf senescence in *Arabidopsis thaliana*. *Plant and Cell Physiolology* **59:** 1753-1764.

[2]**Zhang Y, Liu Z, Wang X, Wang J, Fan K, Li Z, Lin W. 2018.** DELLA proteins negatively regulate dark-induced senescence and chlorophyll degradation in *Arabidopsis* through interaction with the transcription factor WRKY6. *Plant Cell Reports* **37:** 981-992.

[3]**Chen L, Xiang S, Chen Y, Li D, Yu D. 2017.** *Arabidopsis* WRKY45 interacts with the DELLA protein RGL1 to positively regulate age-triggered leaf senescence. *Molecular Plant* **10:** 1174-1189.

[4]**Guo P, Li Z, Huang P, Li B, Fang S, Chu J, Guo H. 2017.** A tripartite amplication loop involving the transcription factor WRKY75, salicylic acid, and reactive oxygen species accelerates leaf senescence. *Plant Cell* **29:** 2854-2870.

5 **Kim HJ, Hong SH, Kim YW, Lee IH, Jun JH, Phee B-K, Rupak T, Jeong H, Lee Y, Hong BS, Nam HG, Woo HR, Lim PO. 2014.** Gene regulatory cascade of senescence-associated NAC transcription factors activated by ETHYLENE-INSENSITIVE2-mediated leaf senescence signaling in *Arabidopsis*. *Journal of Experimental Botany* **65:** 4023-4036.

6 **Huysmans M, Buono RA, Skorzinski N, Radio MC, De Winter F, Parizot B, Mertens J, Karimi M, Fendrych M, Nowack MK. 2018.** NAC transcription factors ANAC087 and ANAC046 control distinct aspects of programmed cell death in the *Arabidopsis* columella and lateral root cap. *Plant Cell* **30:** 2197-2213.

7 **Kuang J-F, Chen J-Y, Luo M, Wu K-Q, Sun W, Jiang Y-M, Lu W-J. 2012.** Histone deacetylase HD2 interacts with ERF1 and is involved in longan fruit senescence. *Journal of Experimental Botany* **63:** 441-454.

8 **Zubo YO, Blakley IC, Franco-Zorrilla JM, Yamburenko MV, Solano R, Kieber JJ, Loraine AE, Schaller GE. 2018.** Coordination of chloroplast development through the action of the GNC and GLK transcription factor families. *Plant Physiology* **178:** 130-147.

9 **Song Y, Yang C, Gao S, Zhang W, Li L, Kuai B. 2014.** Age-triggered and dark-induced leaf senescence require the bHLH transcription factors PIF3, 4, and 5. *Molecular Plant* **7:** 1776-1787.
