# Supplementary material for: ATBS1-INTERACTING FACTOR 2 negatively regulates dark- and brassinosteroid-induced leaf senescence through interactions with INDUCER OF CBF EXPRESSION 1
Source: J Exp Bot. 2019 Nov 30;71(4):1475–90. doi: 10.1093/jxb/erz533 (PMC7031079; doi:10.1093/jxb/erz533)
Supplement: erz533_suppl_Supplementary_Tables_S1-S3 [file erz533_suppl_supplementary_tables_s1-s3.pdf]

Table S1. Primers used in cDNA or promoter amplification of *AIF2*, *ICE1*, *BIN2*, *CBF2,* and *PIF4*

Amplification	Gene fragment cloned	Primer sets (5' to 3')
AIF2FL	Full-length cDNA of *AIF2* coding region	CACCATGGCGTCTCTGATCTCAGATAT
		AATCGGTGGAGGAGCTGAGCCG
AIF2dN	cDNA encoding bHLH and C-terminal domain of *AIF2*	CACCGTTAGTAGCAGCAGCAAC
		AATCGGTGGAGGAGCTGAGCCG
AIF2dC	cDNA encoding N-terminus and bHLH domain of *AIF2*	CACCATGGCGTCTCTGATCTCAGATAT
		CTCCAGAGCCTGAATATAATCAGTTGC
AIF2dbHLH	cDNA encoding N-terminus and a part of bHLH domain of *AIF2*	CACCATGGCGTCTCTGATCTCAGATAT
		CGATCTGTTGCTGCTGCTACTAAC
ICE1FL	Full-length cDNA of *ICE1* coding region	CACCATGGGTCTTGACGGAAAC
		GATCATACCAGCATACCCTGCTG
ICE1dN	cDNA encoding bHLH and C-terminal domain of *ICE1*	CACCGGAGGAGGAAAGGGTAAG
		GATCATACCAGCATACCCTGCTG
ICE1dC	cDNA encoding N-terminus and bHLH domain of *ICE1*	CACCATGGGTCTTGACGGAAAC
		GATCCTTTGTAGAAGTTCCTTCAG
ICE1dbHLH	cDNA encoding N-terminus and a part of bHLH domain of *ICE1*	CACCATGGGTCTTGACGGAAAC
		CTTCTTACCCTTTCCTCCTCCTCC
BIN2	Full-length cDNA of *BIN2* coding region	CACCATGGCTGATGATAAGGAGAT
		AGTTCCAGATTGATTCAAGAA
pCBF2	Promoter region of *CBF2*	CACCCGGCGTCTGAATTTC
		GGTCTCCCCATAAGCAACAAATC
pPIF4	Promoter region of *PIF4*	CACCTTAACCGCCGCAACAAG
		GTTTTATTCTTCAAGTCTTTTGCTATACG

**Table S2.** Primers used in quantitative real-time RT-PCR analysis		
Gene	Locus	Primer set (5′ to 3′)
SAG12	At5g45890	CAGCTGCGGATGTTGTTG
		CCACTTTCTCCCCATTTTG
AtNAP	At1g69490	ATCATGGAAGTAACTTCCCAATC
		TTCAGTTCTTCTCTCTGCTTC
CAB1	At1g29930	AGAGGCCGAGGACTTGCTTTAC
		TATCGGTCCCTTACCAGTGAC
RBCS1A	At1g67090	TCGGATTCTCAACTGTCTGATG
		ATTTGTAGCCGCATTGTCCT
AIF2	At3g06590	AGCAACAGATCGAGAAAACGGAGAG
		CGGTACGGATTGTTTACCGCAAC
BR6ox	At5g38970	GCTGGCCAATATTTGGTGAAACG
		TTGTTGGGCAACCAAGAATGTGA
CPD	At5g05690	GCGGTGTTTTCAGACGTGCAAT
		GAAAGTGCGAGCATCTTTGAAGTGG
CBF1	At4g25490	GCATGTCTCAACTTCGCTGA
		ATCGTCTCCTCCATG CCAG
CBF2	At4g25470	TGACGTGTCCTTATGGAGCTA
		CTGCACTCAAAAACATTTGCA
CBF3	At4g25480	GATGACGACGTATCGTTATGGA
		TACACTCGTTTCTCAGTTTTACAAAC
ACS6	At4g11280	CTGAATCTATTGTCTAAAATCGC
		ACGCATCAAATCTCCACAAAG
SAG15	At5g51070	ATGTCACCTCCATCGCCGCT
		GAACCGTTCGAAAACCGCTG
CCX1	At5g17860	TGACGACCACCGTTCCAAGT
		GCCGTGTCTCCGAGCAAGTA
RbohF	At1g64060	GGTGTCATGAACGAAGTTGCA

		AATGAGAGCAGAACGAGCATCA
GPX4	At2g48150	GTAAACGGTCAAAACGCAGCA
		CCATAACGATCAATCACTAAGCCAT
PIF4	At2g43010	ATCATCTCCGACCGGTTTGC
		AGTGGCTCACCAACCTAGTG
PORC	At1g03630	TAAGAGGCTTAGCGTCAGGATTG
		AGAGAAGCAAACGTGACTCCTGT
BZR1	AT1g75080	CCCATCCCATCTTACCAAGTTAGCC
		GCAACGGTTTCGGGTTCTTAGAAGT
IBH1	At2g06850	GAAGGCTGCGTACGTTTCCA
		CAAGAGGGCTCTGCTCCATA
XTH4	At4g38400	CCTTTGGAACATGTACCAGATCGT
		GGTTGAATGGGAAACGTACTCCTA
EXPL2	At4G38400	TTGTCCTTAGCAGCAGAGCCTTT
		GTAATCGCAAGGAACTCTCCTGTA

**Table S3.** Primers used in ChIP-qPCR analysis		
Gene	Locus	Primer set (5′ to 3′)
UBC30	At5g56150	CAAATCCAAAACCCTAGAAACCGA
		AACGACGAAGATCAACTGGGAA
AIF2 (A)	At3g06590	GCAGCTAGTTAGTCTCATTCC
		GGAACACATCAAGGTATATTAG
AIF2 (B)	At3g06590	CGAATTGGATTCCCACTATAG
		GCAGCCAAATCCTGTTTAAAT
DWF4	AT3g50660	GTGTTTTCTGACTATTGAGGGG
		CGGTACGGTCTCAATCGGTTTA
CBF2	At4g25470	AAAACCGTGGGATCGCTTAG
		TCTTTCTTTTCTAATTCAGG
PIF4	At2g43010	CTTCACTTGTATGTGTCCCACT
		CTCTAGGGACAACAGTACTG
