# Supplementary material for: ATBS1-INTERACTING FACTOR 2 negatively regulates dark- and brassinosteroid-induced leaf senescence through interactions with INDUCER OF CBF EXPRESSION 1
Source: J Exp Bot. 2019 Nov 30;71(4):1475–90. doi: 10.1093/jxb/erz533 (PMC7031079; doi:10.1093/jxb/erz533)
Supplement: erz533_suppl_Supplementary_Data [file erz533_suppl_supplementary_data.pdf]

Article title: ATBS1-INTERACTING FACTOR 2 negatively regulates dark- and brassinosteroid-induced leaf senescence through interactions with ICE1

Authors: Yoon Kim, Seon-U Park, Dong-Min Shin, Giang Pham, You Seung Jeong, Soo-Hwan Kim

The following Supplementary data is available for this article:

Fig. S1. Age-dependent progression of leaf senescence in Col-0, AIF2ox, and *aif2-1* plants.

Fig. S2. Effects of brassinosteroid and its biosynthesis- and signaling-related genetic backgrounds in dark-induced leaf senescence.

Fig. S3. Schematic diagram of PCR-amplified potential BZR1-binding sites (E-box and BRRE) found in promoters of test genes.

Fig. S4. Time-dependent AIF2-Luc expression activity in dark-triggered *pAIF2::AIF2-Luc* plants.

Fig. S5. Antagonistic effects of *bzr1-1D-GFP*-overexpressing *p35S::bzr1-1D-GFP*/Col-0 plants and the full-length AIF2 protein-expressing AIF2ox (*p35S::AIF2FL-EGFP*/Col-0) on dark-triggered leaf senescence.

Fig. S6. *In vivo* interaction test of AIF2 with ICE1 in tobacco.

Fig. S7. Functional analysis of AIF2-ICE1 interaction in regulation of growth-related gene expression.

Fig. S8. Functional analysis of AIF2-ICE1 interaction in tobacco, leading to retardation of dark-induced leaf senescence.

Fig. S9. AIF2/ICE1-dependent expression of *CBF1* and *ACS6* genes in the ICE1FL- or ICE1dC-over-expressing Col-0 or *aif2-1* plants.

Fig. S10. Heatmap and gene ontology classification of PIF4- or non-PIF4-targeted genes in 542 ICE1- and senescence-regulated DEGs.

Table S1. Primers used in cDNA or promoter amplification of *AIF2*, *ICE1*, *BIN2*, *CBF2*, and *PIF4*

Table S2. Primers used in quantitative real-time RT-PCR analysis

Table S3. Primers used in ChIP-qPCR analysis

Table S4. A list of differentially regulated genes grouped according to expression in Col-0 D0, D 2, D5, and *ice1* mutant

Table S5. Transcription factors differentially regulated both in leaves undergoing senescence and in *ice1* mutant
